# The demethylase inhibitor GSK-J4 limits inflammatory colitis by promoting de novo synthesis of retinoic acid in dendritic cells

**DOI:** 10.1038/s41598-020-79122-3

**Published:** 2021-01-14

**Authors:** Cristian Doñas, Jocelyn Neira, Francisco Osorio-Barrios, Macarena Carrasco, Dominique Fernández, Carolina Prado, Alejandra Loyola, Rodrigo Pacheco, Mario Rosemblatt

**Affiliations:** 1 Fundación Ciencia & Vida, Av. Zañartu 1482, 7780272 Ñuñoa, Santiago Chile; 2 Institute of Biomedical Sciences, Faculty of Medicine and Faculty of Life Sciences, Universidad Andres Bello, Av. República 330, Santiago, Chile; 3 Universidad San Sebastián, 7510156 Providencia, Santiago Chile; 4 Departamento de Biología, Facultad de Ciencias, Universidad de Chile, 7800003 Santiago, Chile

**Keywords:** Gastrointestinal diseases, Gastroenterology, Immunology, Adaptive immunity, Immunological disorders, Inflammation, Mucosal immunology

## Abstract

Dendritic cells (DCs) promote T-cell mediated tolerance to self-antigens and induce inflammation to innocuous-antigens. This dual potential makes DCs fundamental players in inflammatory disorders. Evidence from inflammatory colitis mouse models and inflammatory bowel diseases (IBD) patients indicated that gut inflammation in IBD is driven mainly by T-helper-1 (Th1) and Th17 cells, suggesting an essential role for DCs in the development of IBD. Here we show that GSK-J4, a selective inhibitor of the histone demethylase JMJD3/UTX, attenuated inflammatory colitis by reducing the inflammatory potential and increasing the tolerogenic features of DCs. Mechanistic analyses revealed that GSK-J4 increased activating epigenetic signals while reducing repressive marks in the promoter of retinaldehyde dehydrogenase isoforms 1 and 3 in DCs, enhancing the production of retinoic acid. This, in turn, has an impact on regulatory T cells (Treg) increasing their lineage stability and gut tropism as well as potentiating their suppressive activity. Our results open new avenues for the treatment of IBD patients.

## Introduction

To avoid inflammatory reactions in the absence of threats T-cells reactive to self or foreign-innocuous antigens have to be eliminated or rendered tolerant by acquiring a phenotype with suppressive functions (i.e., regulatory T-cells; Treg), a process in which dendritic cells (DCs) play a fundamental role. Indeed, constitutive depletion of DCs leads to spontaneous fatal autoimmunity^1^. Conversely, during the onset of inflammatory and autoimmune disorders, DCs seem to be critical for the induction of inflammatory phenotypes on T-cells reactive to innocuous-antigens^2^. This dual potential of DCs of promoting tolerance or inducing inflammation to innocuous-antigens makes these cells fundamental players in the physiopathology of inflammatory disorders.

Gut mucosa constitutes one of the primary tissues in which immune tolerance is actively induced to foreign-innocuous antigens, for instance, those coming from food and commensal microbiota. Thus, the failure of tolerogenic mechanisms associated to gut-mucosa results in inflammatory bowel diseases (IBD), a group of chronic remittent inflammatory disorders of the gastrointestinal tract, among which Crohn’s disease and ulcerative colitis are the most common. Evidence from inflammatory colitis mouse models and IBD patients has indicated that gut inflammation in IBD is driven mainly by T-helper-1 (Th1) and Th17 cells^3^, thus suggesting an essential role for DCs in the development of IBD.

Kruidenier et al. demonstrated that GSK-J4, a selective histone demethylase inhibitor, attenuates the production of proinflammatory cytokines by macrophages^4^. Indeed, it was recently demonstrated that GSK-J4 inhibits the activation of the NLRP3 inflammasome in macrophages, thus attenuating the inflammatory response mediated by innate immunity in a mouse model of inflammatory colitis^5^. Nevertheless, we have recently shown that GSK-J4 induces a tolerogenic phenotype in DCs, increasing the generation and the potency of Treg and thereby attenuating the manifestation of autoimmunity in the central nervous system^6^. Accordingly, here we studied how this drug’s therapeutic effect depends on DCs in a mouse model of inflammatory colitis.

## Results

### Selective inhibition of the histone demethylase JMJD3/UTX ameliorates the manifestation of inflammatory colitis

**Figure 1:**
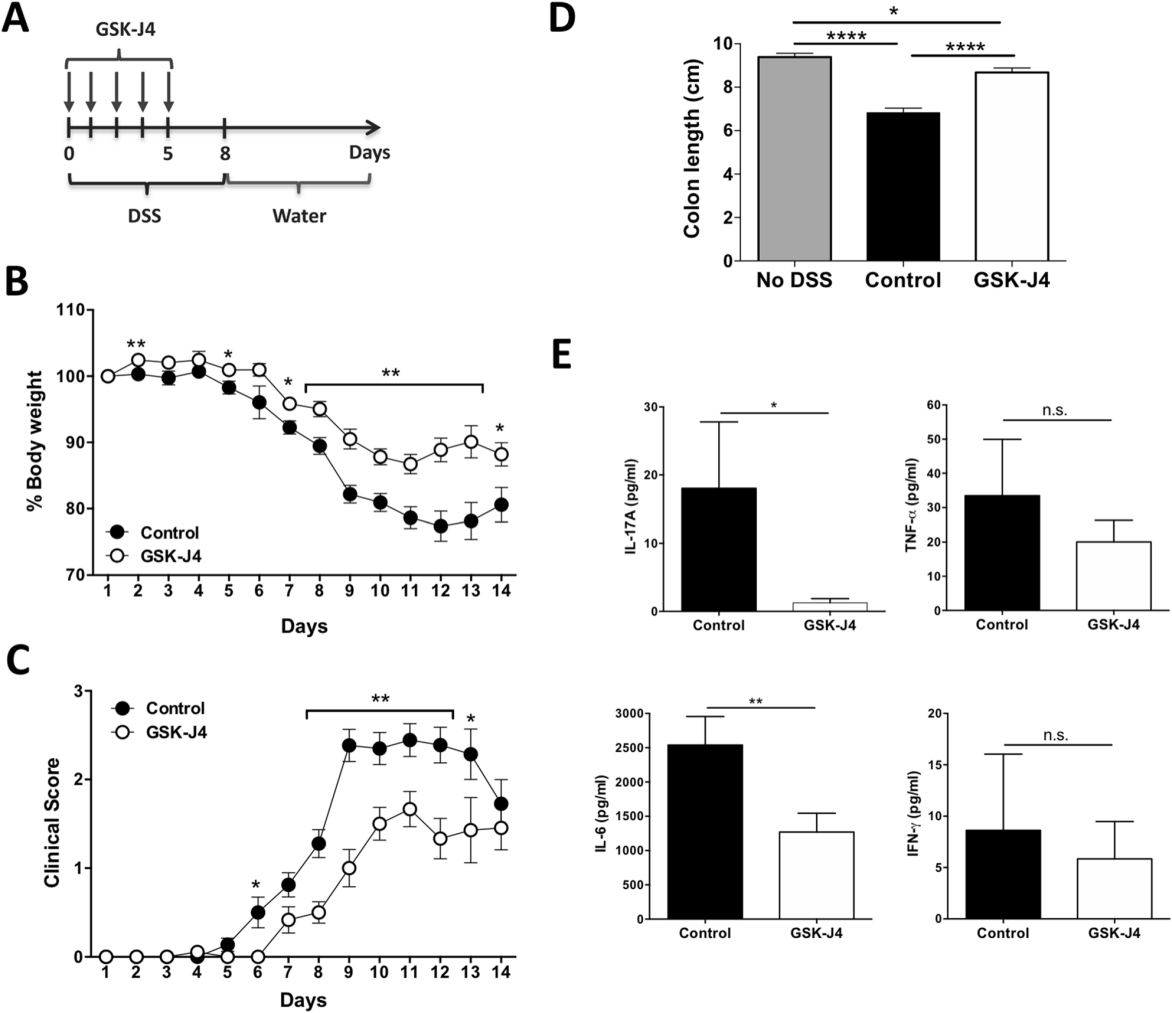
GSK-J4 treatment ameliorates DSS-induced acute colitis. Colitis was induced in C57BL/6 mice by the administration of 1.5% DSS. Vehicle or GSK-J4 (1 mg/kg) was administered daily for 5 days starting the same day DSS treatment began. (**A**) Scheme illustrating the experimental design. (**B**) Bodyweight change respect to the initial weight was calculated throughout the time-course of disease development. (**C**) Clinical score throughout the time-course of disease development. (**D**) Colon length of each experimental group determined on day 14 after colitis induction. (**B**–**D**) Values represent mean ± SEM from 18–22 (**B**,**C**) or 11–12 (**D**) mice per group. (**E**) At the peak of disease severity (day 12), mice were sacrificed and colon slices were cultured in fresh medium for 24 h and then the supernatant was evaluated for the secretion of IL-17, IL-6, TNFα, and IFNγ by CBA. Values represent mean ± SEM from 5 to 9 mice per group. *p < 0.05; **p < 0.01; ***p < 0.001; ***p < 0.0001 as determined by Student’s t-test (**B**,**C**,**E**) or one-way ANOVA followed by Tukey’s post-hoc test (**D**). n.s., not significant differences were found.

To evaluate the therapeutic potential of GSK-J4, we employed the mouse model of inflammatory colitis induced by the administration of dextran sodium sulphate (DSS; Fig. 1A). Importantly, the treatment with GSK-J4 reduced the loss of bodyweight and morbidity in DSS-treated mice (Fig. 1B, C). The lessening of disease manifestation was associated with decreased gut inflammation as determined by attenuation of colon shortening (Fig. 1D) and by reduced production of the inflammatory cytokines IL-6 and IL-17 without affecting TNF-α and IFN-γ production in gut mucosa (Fig. 1E), suggesting a selective inhibition of the Th17 response. To evaluate whether the therapeutic effect of the drug involved changes in the frequency of inflammatory and suppressive subsets of CD4^+^ T-cells, we induced colitis by the administration of DSS in the drinking water in mice treated with GSK-J4 or vehicle. At the peak of disease manifestation (day 12 post-induction) we isolated mononuclear cells from the colonic lamina propria (cLP), mesenteric lymph nodes (MLN) and spleen and analysed the functional phenotypes of CD4^+^ T-cells. Whereas GSK-J4 had no effect in Treg and Th1 frequencies infiltrating the cLP, we observed that the drug clearly reduced Th17 frequency in the gut-mucosa (Fig. S1A–C). Since IL-10 produced by Treg is fundamental in maintaining tolerance, particularly at intestinal tissues^7^, we also analysed the frequency of IL-10 producing CD4^+^ T-cells in the cLP. The results showed that systemic GSK-J4 administration enhanced the frequency of IL-10-producing CD4^+^ T-cells in the cLP, without affecting IL-10 production by CD4^+^ T-cells from MLN and spleen (Fig. S1D). Interestingly, the analysis of Foxp3 expression in IL-10^+^ CD4^+^ T-cells revealed that systemic GSK-J4 increased the frequency of Foxp3^−^ IL-10^+^ CD4^+^ T-cells (a phenotype resembling the Tr1 suppressive lymphocytes^8^), but not of Foxp3^+^ IL-10^+^ CD4^+^ T-cells (Fig. S1E,F). Thus, these results suggest that systemic GSK-J4 administration increases the frequency of Tr1 cells and attenuates Th17 response in the colonic mucosa upon gut inflammation. To gain a deeper insight into the mechanism underlying the effect of GSK-J4 in IL-10 production by T-cells upon DSS-induced colitis, we performed similar experiments but analysed the functional phenotypes of T-cells at an earlier time-point (day eight post-induction). Despite the fact that we observed a trend of increasing IL-10 production by colonic Foxp3^+^ Treg cells upon GSK-J4 treatment, no significant differences were observed in any T-cell phenotype when compared GSK-J4-treated animals with the control group at this time point (Fig. S2A,B). Similarly, we did not observe any difference in the secretion of inflammatory or anti-inflammatory cytokines produced by the colonic mucosa of GSK-J4-treated mice compared with control animals when analysed at day eight after colitis induction (Fig. S3). These results suggest that the therapeutic effect observed for GSK-J4 in inflammatory colitis involves an increased production of IL-10 by T-cells at the peak of disease manifestation in the gut mucosa.

Since the DSS-induced colitis model depends on innate and adaptive immunity, we next aimed to analyse the therapeutic effect of GSK-J4 in gut inflammation in the absence of adaptive immunity. To this end, we used animals deficient in *rag1* gene, which codify for the recombination activating gene 1, an enzyme fundamental for the development of T and B lymphocytes. Interestingly, although we did not observe any difference in the production of inflammatory or anti-inflammatory cytokines in the colonic mucosa between both experimental groups, the systemic administration of GSK-J4 induced a significant attenuation of the bodyweight loss in *rag1*^−/−^ mice upon DSS-induced inflammatory colitis (Fig. S4). These results suggest that the therapeutic effect of the drug is, at least in part, dependent on innate immunity. Since previous studies have indicated that GSK-J4 reduces macrophages inflammatory response^4,5^, we next sought to confirm the anti-inflammatory effect of GSK-J4 in macrophages on our experimental system. In agreement with previous studies, we observed that systemic GSK-J4 administration induced a reduction in the production of TNFα by macrophages upon DSS-induced colitis (Fig. S5). Innate lymphoid cells (ILCs) have been found to be abundant in the intestinal mucosa, where they play an important role regulating gut homeostasis^9^. Accordingly, we also determined the effect of GSK-J4 on this subset of innate cells upon DSS-induced gut inflammation. Our results show that systemic administration of GSK-J4 selectively reduced the frequency of ILCs in the colonic mucosa, but not in the small intestine lamina propria (Fig. S6A,B). Interestingly, this effect was not due to alterations in the production of IFN-γ or IL-10 by ILCs (Fig. S6B). Altogether, these results indicate that the therapeutic effect exerted by the systemic administration of GSK-J4 is, at least in part, due to an anti-inflammatory effect on the innate immune system. However, the potential role of GSK-J4 on the adaptive immunity in gut inflammation remains poorly explored.

### The selective inhibition of the histone demethylase JMJD3/UTX in DCs ex vivo reduces the severity of inflammatory colitis

**Figure 2:**
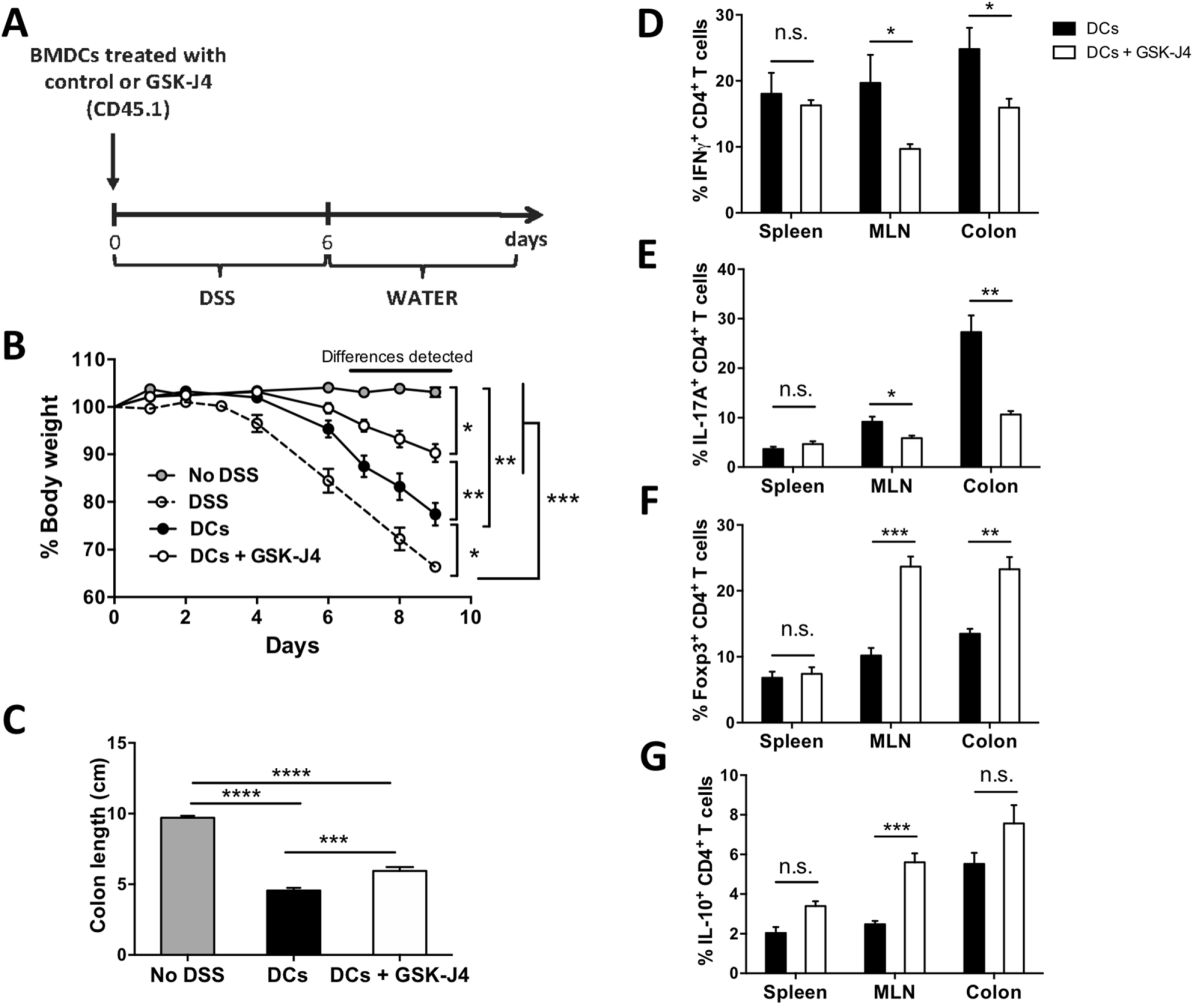
DCs treated ex vivo with GSK-J4 ameliorate DSS-induced acute colitis. Bone marrow-derived DCs obtained from *Cd45.1*^+/+^ *Cd45.2*^−/−^ mice were treated with vehicle (control) or with 25 nM GSK-J4 for 18 h, and then 3 × 10^6^ DCs/mice were i.v. transferred into wild-type *Cd45.1*^−/−^ *Cd45.2*^+/+^ recipient mice. Colitis was immediately induced in recipient mice by treatment with 1.5% DSS for 6 days. (**A**) Scheme illustrating the experimental design. (**B**) Bodyweight change respect to the initial weight was calculated throughout the time-course of the disease. Values represent mean ± SEM from 4 to 10 mice per group. (**C**) Colon length of each experimental group was evaluated on day 9 after colitis induction. Values represent mean ± SEM from 8 to 10 mice per group. (**B**,**C**) As a control, a group of mice did not receive DSS treatment. (**D**–**G**) At the peak of disease severity (day 9), mice were sacrificed and mononuclear cells were isolated from spleen, MLN and colon followed by ex vivo stimulation with PMA/ionomycin in the presence of brefeldin A. Intracellular cytokine staining analyses were carried out by flow cytometry. Frequency of (**D**) IFNγ^+^, (**E**) IL-17A^+^, (**F**) Foxp3^+^, and (**G**) IL-10^+^ cells from CD4^+^ T-cells isolated from spleen, MLN and colon. Values represent mean ± SEM from 5 mice per group. *p < 0.05; **p < 0.01; ***p < 0.001 as determined by one-way ANOVA followed by Tukey’s post-hoc test (**B**,**C**) or two-way ANOVA followed by Bonferroni’s post-hoc test (**D**–**G**). n.s., not significant differences were found.

Since we previously found that GSK-J4 increases the tolerogenic function of DCs through the generation of Treg with higher suppressive activity in a mouse model of multiple sclerosis^6^, we wondered whether this drug exerts a direct action on DCs in the context of gut inflammation. To address this possibility, we next performed experiments in which mice undergoing colitis received the intravenous transfer of DCs treated with GSK-J4 ex vivo (Fig. 2A). The results show that mice receiving the transfer of GSK-J4-treated DCs displayed an attenuated manifestation of inflammatory colitis in comparison with those mice receiving untreated DCs, as determined by a reduced bodyweight loss and a lessened shortening of colon length (Fig. 2B, C). Furthermore, we observed decreased frequencies of the Th1 and Th17 inflammatory CD4^+^ T-cell subsets and an increased percentage of Treg in the cLP and MLN of mice receiving GSK-J4-treated DCs in comparison with those animals receiving untreated DCs (Fig. 2D–F). Interestingly, although systemic treatment of mice with GSK-J4 increased the frequency of IL-10-producing CD4^+^ T-cells in the cLP without any effect on the percentage of Treg infiltrating cLP (Fig. S1), mice receiving GSK-J4-treated DCs presented a higher percentage of Treg with no effect on the frequency of IL-10-producing CD4^+^ T-cells in cLP (Fig. 2F, G). This difference may be due to the action of systemic GSK-J4 on innate immune cells, an effect that is avoided when DCs are incubated ex vivo with the drug and then transferred into DSS-treated mice.

According to the prominent anti-inflammatory effect exerted by GSK-J4 on DCs in the context of inflammatory colitis, we next aimed to decipher the underlying mechanism. Importantly, retinoic acid (RA), which is especially abundant in gut-associated lymphoid tissues (GALT), exerts a potent effect inducing Treg differentiation and IL-10 production^10^. Moreover, in contrast to DCs from other sources, DCs coming from GALT express retinaldehyde dehydrogenase (RALDH)^11^, which allows these cells to synthesize RA using as substrate vitamin A contained in food^12^. By producing RA, GALT-DCs induces the up-regulation of gut-homing receptors CCR9 and α4β7 in activated CD4^+^ T-cells, imprinting gut-tropism in these cells^13^. Thus, in the absence of inflammatory cues, GALT-DCs present antigens to naïve CD4^+^ T-cells in MLN inducing antigen-specific differentiation of these naïve T-cells into Treg with gut-tropism, thereby promoting oral tolerance^14^. Since RA is known to improve the tropism of DC to gut tissue, we first hypothesized that GSK-J4 might also improve DCs tropism for GALT. Accordingly, we analysed if the ex vivo treatment of DCs with GSK-J4 affected the extent of DCs infiltration in different tissues of mice undergoing inflammatory colitis. The results showed that treatment of DCs with GSK-J4 did not affect their infiltration in the spleen, MLN or cLP in DSS-treated mice (Fig. S7), thus ruling out the possibility that the anti-inflammatory effect of this drug was due to increased infiltration of DCs in GALT.

### The selective inhibition of the histone demethylase JMJD3/UTX in DCs increases RALDH expression and activity in these cells by enhancing the mark H3K4me3 and decreasing H3K27me3 on the *raldh1* and *raldh3* promoters

**Figure 3:**
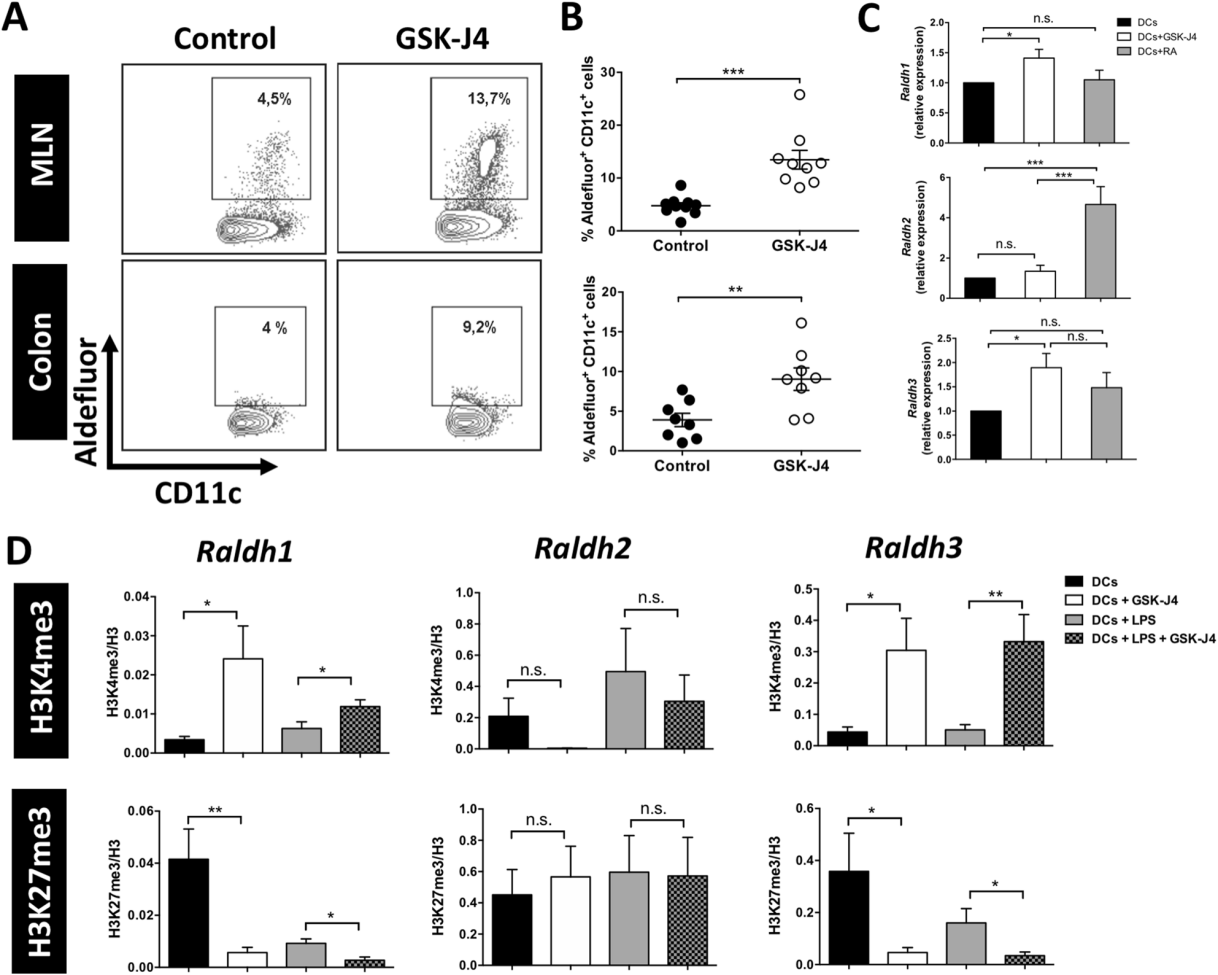
GSK-J4 increases RALDH activity and expression in DCs by enriching the mark H3K4me3 and decreasing H3K27me3 on the *raldh1* and *raldh3* promoters. (**A**) Representative dot-plot of RALDH activity using Aldefluor assays in DCs isolated at day 12 from the colonic lamina propria (colon) and MLN of mice treated as described in Fig. 1A. Numbers represent the frequencies of cells in the corresponding quadrant. (**B**) Frequencies of Aldefluor^+^ CD11c^+^ cells from at least six animals per group. (**C**,**D**) Splenic CD11c^+^ DCs from C57BL/6 mice were treated with 25 nM GSK-J4 or 10 nM RA for 16 h. (**C**) RT-qPCR analysing *raldh1* (top panel), *raldh2* (middle panel), and *raldh3* (bottom panel) expression were performed on DCs. Relative expression levels were normalized using 18S RNA as control. (**D**) DCs were either unstimulated or stimulated with 100 ng/mL LPS in the presence or absence of GSK-J4 for 16 h. Chromatin Immunoprecipitation (ChIP) assays were carried out using specific antibodies to H3K4me3 (top panels), H3K27me3 (bottom panels). Association of H3K27me3 or H3K4me3 to the promoters of *raldh1* (left panels), *raldh2* (middle panels), and *raldh3* (right panels) was quantified by qPCR by using specific primers. PCR products were normalized to the input DNA and histone H3 levels. Values represent mean ± SEM from six independent experiments. *p < 0.05; **p < 0.01, ***p < 0.001 as determined by Student’s t-test (**B**) or one-way ANOVA followed by Tukey’s post-hoc test (**C**,**D**). n.s., not significant differences were found.

**Figure 4:**
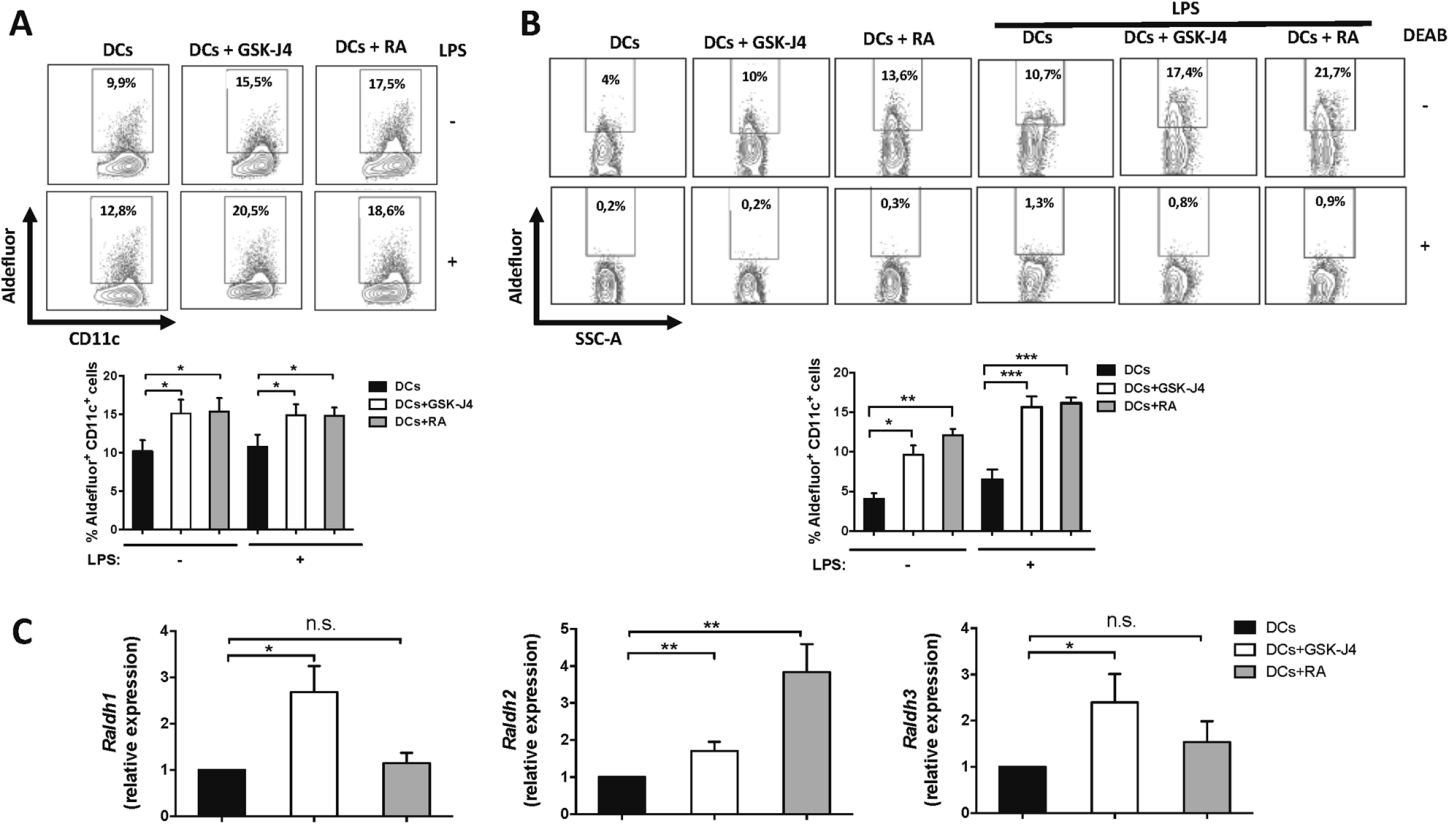
GSK-J4 promotes RALDH activity in DCs isolated from spleen or MLN. (A-B) CD11c^+^ DCs were isolated from the MLN (**A**) and the spleen (**B**) and either unstimulated or stimulated with 100 ng/mL LPS in the presence or absence of 25 nM GSK-J4 or 10 nM RA for 16 h. Top panels show representative dot-plots of RALDH activity using Aldefluor assay. Numbers represent the frequencies of cells in the corresponding quadrant. Bottom panels show the frequency of Aldefluor^+^ cells from the CD11c^+^ gate obtained from at least six independent experiments. (**C**) Splenic CD11c^+^ DCs from C57BL/6 mice were stimulated with 100 ng/mL LPS in the presence or absence of 25 nM GSK-J4 for 16 h. RT-qPCR analysing *raldh1* (left panel), *raldh2* (middle panel), and *raldh3* (right panel) expression were performed on DCs. Relative expression levels were normalized using 18S RNA as control. Data represent mean ± SEM from six independent experiments. *p < 0.05; **p < 0.01; ***p < 0.001 as determined by one-way ANOVA followed by Tukey’s post-hoc test (**A**–**C**). n.s., not significant differences were found.

We next analysed whether GSK-J4 has an impact on the production of RA by GALT-DCs and thus an increase of IL-10 by CD4^+^ T cells. These analyses indicated that, indeed, GSK-J4 treatment promoted a robust increment in RALDH-activity in MLN DCs. The results also suggest a higher RALDH-activity on those DCs infiltrating the cLP upon GSK-J4 treatment (Fig. 3A,B). Of note, this effect was observed at the peak of DSS-induced colitis (day 12), but not at an earlier time point (day 8; figure S2C,D). A similar effect potentiating RALDH-activity was observed in DCs isolated from MLN or spleen and treated ex vivo with GSK-J4 either in the absence or in the presence of an inflammatory stimulus (Fig. 4A, B). Since there are three isoforms of RALDH which display distinct substrate affinities and present differential expression in some cell types^15–17^, we next analysed the effect of GSK-J4 on the expression of the different RALDH isoforms in DCs and compared this to the direct effect of RA. Interestingly, our results show that GSK-J4 induced *raldh1* and *raldh3* transcription, while it had a very week effect in the levels of *raldh2* transcripts. Conversely, the effect of RA was confined to *raldh2* transcription (Fig. 3C). Similar results were observed in the presence of LPS (Fig. 4C). Taken together, these results suggest a complementary effect of GSK-J4 and RA, in promoting a tolerogenic potential in DCs.

Since GSK-J4 is a known specific inhibitor of the histone H3 lysine-demethylase JMJD3^18^, we next analysed the effect of the drug on the level of methylation of histones in the different promoters of *raldh* in DCs. For this purpose, we determined the extent of tri-methylation of histone H3 both at lysine 4 (H3K4me3) and lysine 27 (H3K27me3), which have been described to exert permissive and repressive effects respectively in gene transcription^19^. Consistent with the selective effect of GSK-J4 in inducing the transcription of *raldh1* and *raldh3*, the treatment of DCs with this drug increased the degree of the permissive mark H3K4me3 in the promoters of *raldh1* and *raldh3*, but without effect on the *raldh2* promoter (Fig. 3D). Conversely, GSK-J4 greatly reduced the levels of the repressive mark H3K27me3 in the promoters of *raldh1* and *raldh3*, with no effect on the *raldh2* promoter (Fig. 3D). Thus, these results unravel the molecular mechanism exerted by GSK-J4 favouring a tolerogenic behaviour on DCs by acting at the level of the epigenetic modifications on the *raldh1* and *raldh3* promoters.

### The selective inhibition of the histone demethylase JMJD3/UTX increases de novo synthesis of RA by DCs

**Figure 5:**
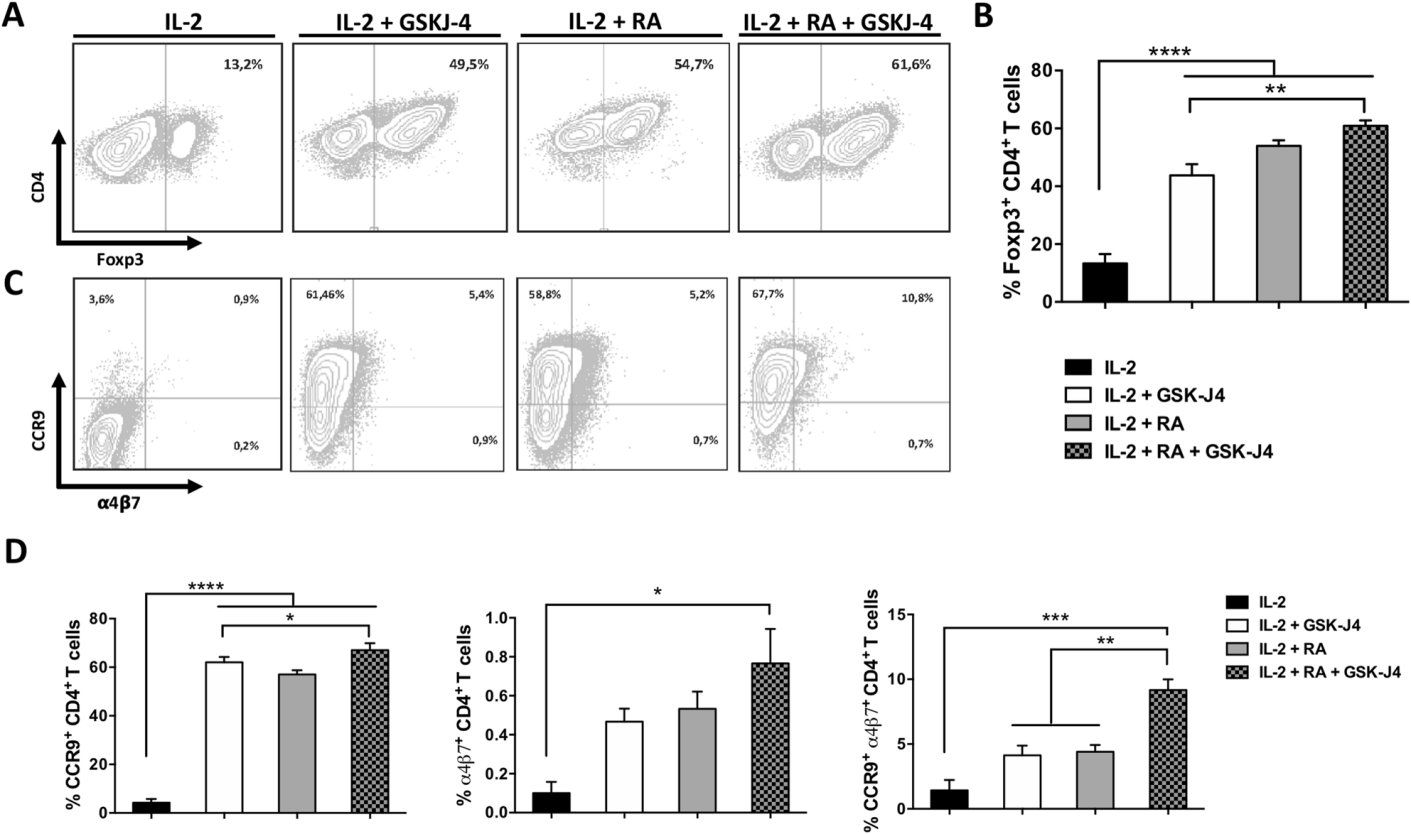
GSK-J4 promotes RA synthesis by DCs. Naïve CD4^+^ CD25^−^ Foxp3^−^ T cells were isolated from *Foxp3*^*GFP*^ mice by cell-sorting and activated with splenic DCs and anti-CD3 Ab under standard Treg polarising conditions (IL-2), or in the presence of either 25 nM GSK-J4, 10 nM RA or both for 4 days. (**A**,**B**) The extent of iTreg generation was evaluated as the frequency of GFP^+^ (Foxp3^+^) cells in the CD4^+^ population. (**A**) Representative dot-plots and (**B**) the quantification are shown. (**C**,**D**) CCR9 and α4β7 expression was evaluated in CD4^+^ T cells. (**C**) Representative dot-plots and (**D**) the quantification are shown. Data represent mean ± SEM from six independent experiments. *p < 0.05; **p < 0.01; ***p < 0.001; ****p < 0.0001 as determined by one-way ANOVA followed by Tukey’s post-hoc test (**A**–**C**). n.s., not significant differences were found.

Because our results indicated a complementary role of RA and GSK-J4 in promoting the tolerogenic potential of DCs, we next evaluated whether GSK-J4 was able to potentiate the effect of RA in the differentiation of Treg with gut-tropism. To this end, DCs treated with GSK-J4, RA, or both, were co-cultured with naïve CD4^+^ T-cells and the acquisition of gut-tropism and Treg-phenotype were determined. RA increased the extent of Treg differentiation (Fig. 5A,B), as described before^20^. Similarly, GSK-J4 promoted Treg generation and, indeed, we observed an additive effect of both RA and GSK-J4 in the acquisition of a Treg-phenotype (Fig. 5A,B). As expected, RA enhanced the expression of gut-homing receptors α4β7 and CCR9 (Fig. 5C,D)^21^. A similar increase was found when naïve T cells were incubated with GSK-J4, probably as a consequence of the production of RA by DCs under the influence of the drug. The combination of RA plus GSK-J4 had a slight but noticeable additive effect on the expression of gut-homing receptors (Fig. 5C,D). Importantly, we observed that the higher extent of Treg differentiation induced by the effect of GSK-J4 on DCs was dependent on the stimulation of the RA receptor (RAR), as the pre-treatment of DCs with a RAR-antagonist (LE135) abrogated the GSK-J4 effect on Treg generation (Fig. S8).

To evaluate the possibility that GSK-J4 may also exert a direct effect on T-cells favouring gut-tropism and acquisition of a Treg-phenotype, we activated naïve CD4^+^ T-cells with anti-CD3 and anti-CD28 antibodies (Abs) under Treg polarising conditions, in the absence of DCs. Treg generated under these conditions were then treated with GSK-J4, RA or both together. These results show that neither RA nor GSK-J4, alone or jointly, increased the generation of Treg significantly (Fig. S9). These results differ from those obtained previously by Mucida and colleagues^22^. The different outcome of our results might be due to the fact that our iTreg standard polarizing conditions included not only TGF-β and IL-2, but also anti-IL-4 and anti-IFN-γ Abs, whilst Mucida and colleagues used only TGF-β and IL-2. Since the iTreg polarizing conditions used here were more restrictive, we observed ≈ 50% iTreg conversion under basic standard conditions (Fig. S9) and we did not observe a further increase when treated with RA or GSK-J4. On the other hand, in the absence of DCs, only RA, but not GSK-J4 induced the expression of gut-homing receptors. Moreover, GSK-J4 had no effect in potentiating the effect of RA in the imprinting of gut-tropism in T-cells (Fig. S9). Thus, these results rule out the contribution of GSK-J4 by acting directly on T-cells reinforcing the view that the potent anti-inflammatory effect exerted by GSK-J4 is due to a direct action on DCs promoting a tolerogenic behaviour on these cells.

### GSK-J4 increases the suppressive activity and lineage stability of Treg upon gut inflammation

**Figure 6:**
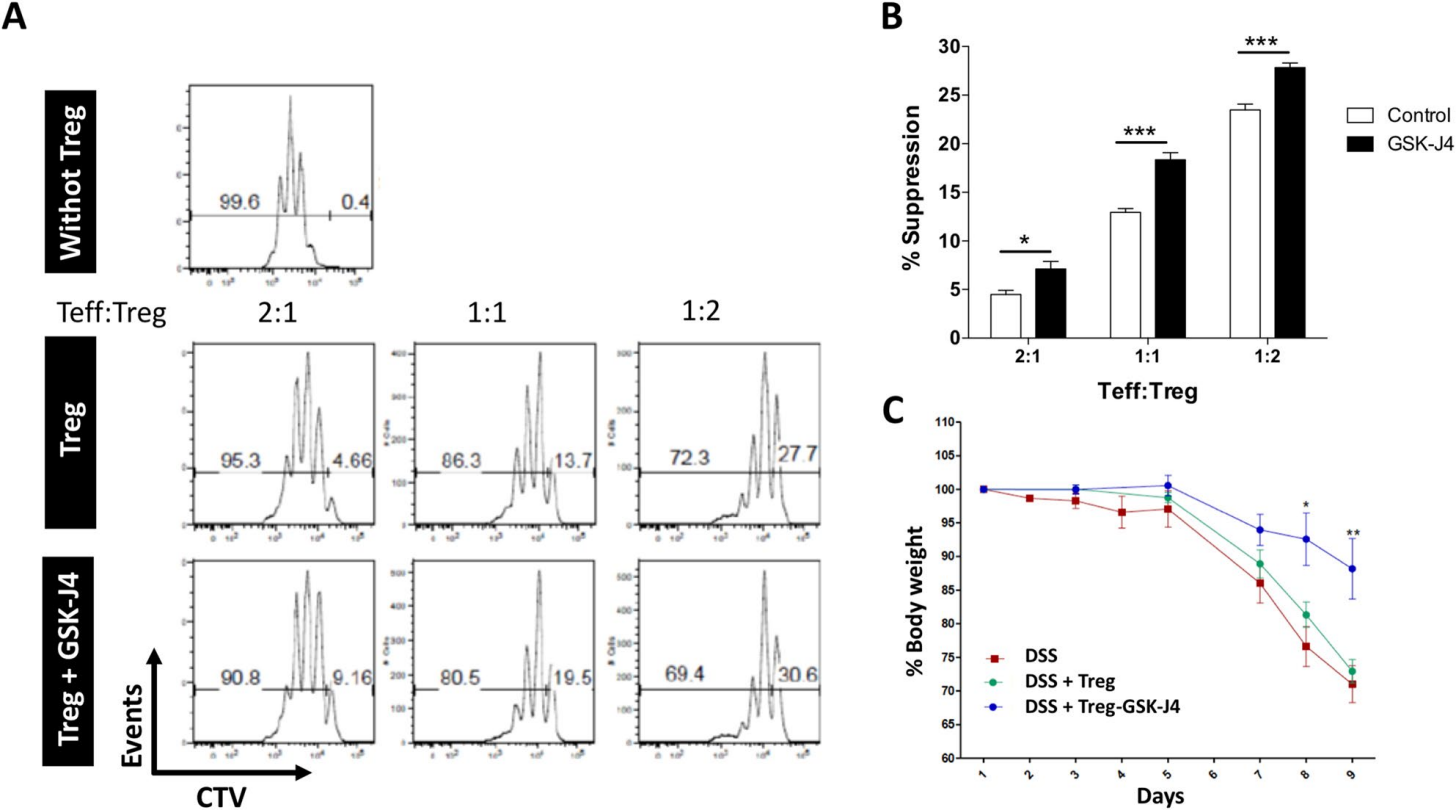
DCs exposed to GSK-J4 generate Treg with higher suppressive activity. Naïve CD4^+^ CD25^−^ Foxp3^−^ T cells were isolated from *Foxp3*^*GFP*^ mice by cell-sorting and activated with splenic DCs and anti-CD3 Ab under standard Treg polarising conditions in the absence (Control) or the presence of GSK-J4 25 nM. After 5 days of culture, GFP^+^ Treg cells were purified by cell-sorter and their suppressive activity was analysed in vitro (**A**,**B**) and in vivo (**C**). (**A**,**B**) Effector CD4^+^ CD25^−^ T cells (Teff) were loaded with 5 μM CTV and activated with DCs and anti-CD3 Ab in the presence of Treg (at different Teff:Treg ratios). Three days later, Teff proliferation was quantified as the dilution of CTV-associated fluorescence by flow cytometry. As a control to determine the maximal Teff proliferation, Teff were activated in the absence of Treg (without Treg). (**A**) Representative histograms of CTV dilution profiles are shown. Markers show the population of Teff displaying CTV dilution (left markers) and Teff displaying no CTV dilution (right markers). The percentages of cells covered by each marker are indicated. (**B**) The extent of suppression is quantified as the percentage of inhibition of Teff proliferation relative to maximal proliferation (without Treg). Data represent mean ± SEM from three independent experiments. (**C**) Treg were i.v. transferred (7.5 × 10^5^ cells per mouse) into WT recipient mice which simultaneously received 1.5% DSS during 8 days. Bodyweight changes respect to the initial weight was calculated throughout the time-course of disease development. Data represent mean ± SEM from 4 mice per group. *p < 0.05; **p < 0.01; ***p < 0.001 when Treg and Treg + GSK-J4 groups are compared as determined by Student’s t-test (**B**,**C**).

To evaluate whether GSK-J4 has an impact on the potency of Treg function, we next analysed the suppressive activity of Treg generated in the presence of this drug. For this purpose, Treg were differentiated in the presence of DCs treated or not with GSK-J4 and then their suppressive activity was determined as the ability to reduce the proliferation of effector T cells in vitro. The results show that Treg generated in the presence of GSK-J4 display a higher suppressive activity than Treg generated in the absence of the drug (Fig. 6A,B). To determine the relevance of this effect in vivo in the context of gut inflammation, we next generated Treg in the presence of GSK-J4 or vehicle and then were i.v. transferred into recipient mice in which inflammatory colitis was previously induced by DSS. The results show that mice receiving Treg generated with GSK-J4 developed a less severe disease manifestation than those mice receiving the transfer of Treg generated in the absence of the drug (Fig. 6C). Thus, these results suggest that Treg generated in the presence of GSK-J4 exert a stronger suppressive activity in vivo attenuating gut inflammation.

**Figure 7:**
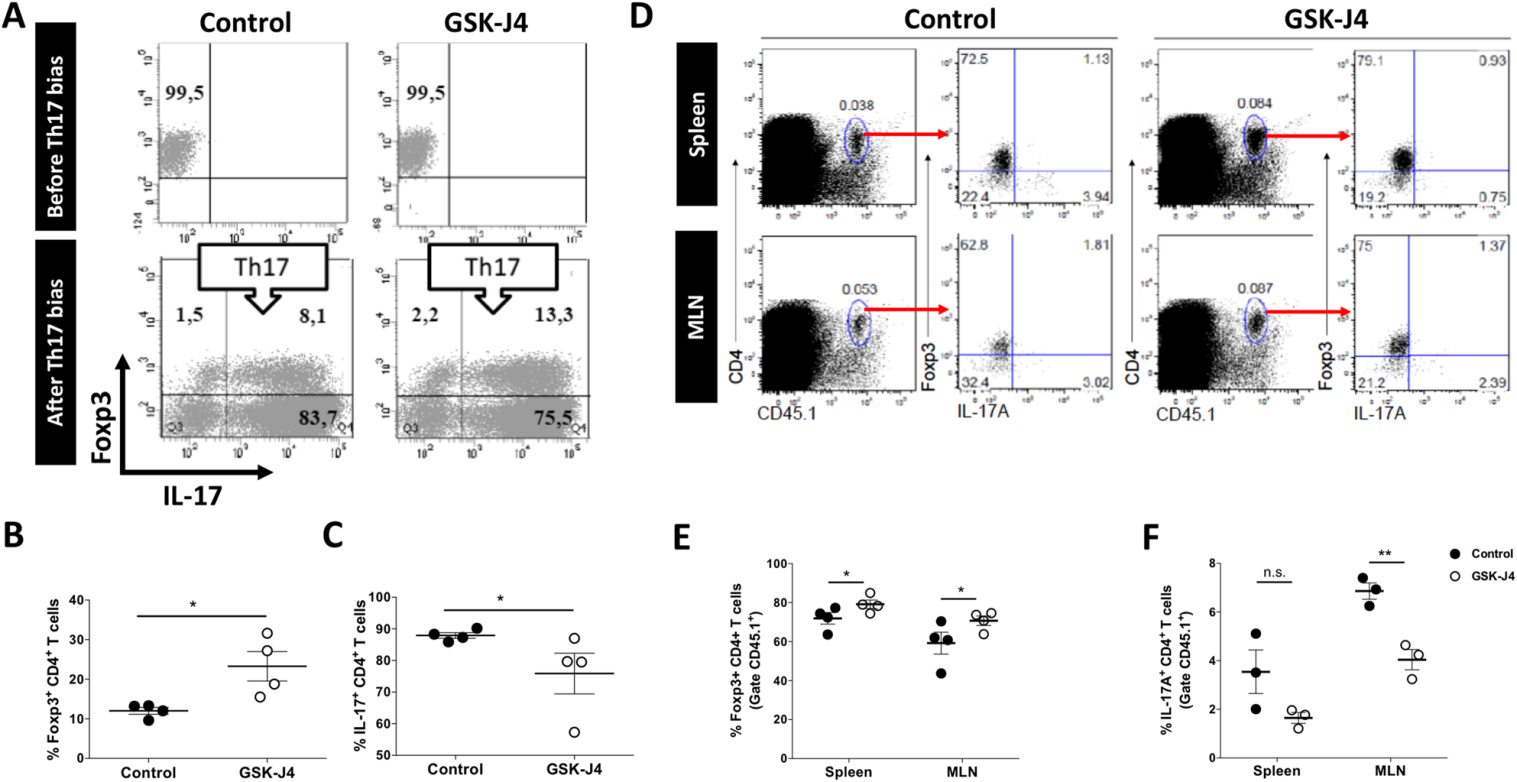
DCs exposed to GSK-J4 generate Treg with higher lineage stability. Naïve CD4^+^ CD25^−^ Foxp3^−^ T cells were isolated from *Foxp3*^*GFP*^* Cd45.1*^+/+^ *Cd45.2*^−/−^ mice by cell-sorting and activated with splenic DCs and anti-CD3 Ab under standard Treg polarising conditions in the absence (Control) or the presence of GSK-J4 25 nM. After 5 days of culture, GFP^+^ CD4^+^ Treg cells were purified by cell-sorter. (**A**–**C**) Treg were incubated in Th17 biased conditions for 5 days and the level of Foxp3 and IL-17A expression was evaluated by flow cytometry. (**A**) Representative dot-plots of Treg before (top panels) or after (bottom panels) incubation under Th17 biased conditions. Numbers on the dot-plots indicate the percentage of cells in each quadrant. (**B**–**C**) Quantification of the Foxp3^+^ (**B**) and IL-17A^+^ (**C**) frequency in the CD4^+^ T cell population. Each symbol represents data obtained from a single mouse (n = 4 per group). Mean ± SEM are shown. (**D**–**F**) Treg were i.v. transferred (7.5 × 10^5^ cells per mouse) into WT *Cd45.1*^−/−^ *Cd45.2*^+/+^ recipient mice which 24 h later received 1.5% DSS during 8 days. On day 10 after colitis induction, cells from the spleen and MLN were isolated and the extent of Foxp3 and IL-17A expression was analysed in the CD4^+^ CD45.1^+^ population by flow cytometry. (**D**) Representative dot-plots of CD4 versus CD45.1 and of Foxp3 versus IL-17A are shown. Numbers on the dot-plots indicate the percentage of cells in each region or quadrant. Red arrows indicate the gate analysed. (E–F) Quantification of the Foxp3^+^ (**E**) and IL-17A^+^ (**F**) frequency in the CD4^+^ CD45.1^+^ population. Each symbol represents data obtained from a single mouse (n = 3–4 per group). Mean ± SEM are shown. *p < 0.05; **p < 0.01 as determined by Student’s t-test (**B**,**C**) or two-way ANOVA followed by Sidak’s post-hoc test (**E**,**F**). n.s., not significant differences were found.

Several studies have indicated that Treg might transdifferentiate into the Th17 inflammatory phenotype under inflammatory conditions, including gut inflammation^23,24^. Since we have previously demonstrated that Treg generated with GSK-J4 display a reduced phenotypic plasticity^6^, we wondered whether GSK-J4 affects Treg stability upon inflammatory colitis. As a first approach, we generated Treg in the presence of GSK-J4 or just vehicle and then they were exposed to Th17-biased conditions in vitro. The results show that Treg generated in the presence of the drug maintained a higher extent of Foxp3 expression (Fig. 7A,B) and acquired a lower ability to produce IL-17A (Fig. 7A,C), indicating a higher phenotypic stability and lower degree of plasticity. As a second approach to address the impact of GSK-J4 in Treg stability, Treg were generated in vitro in the presence of GSK-J4 or vehicle and then transferred into mice treated with DSS. Ten days later, the phenotype of transferred Treg was analysed in the MLN and the spleen of recipient mice. The results show that when Treg are generated in the presence of GSK-J4, these cells retain Foxp3 expression in a higher degree in both spleen and MLN (Fig. 7D,E). Conversely, GSK-J4 resulted in the reduced acquisition of the Th17 phenotype under intestinal inflammation (Fig. 7D,F). Altogether these results indicate that Treg generated in the presence of GSK-J4-treated DCs acquire higher lineage stability.

## Discussion

Collectively this study demonstrates that GSK-J4, a selective inhibitor of the histone demethylase JMJD3/UTX, attenuates DSS-induced acute colitis. Mechanistic analysis shows that this effect is mediated by changes on histone post-translational modifications at the *raldh1* and *raldh3* promoters in DCs, which increases RALDH activity in these cells, thus favouring the de novo RA synthesis. This in turn induces Treg with higher suppressive activity and stability as well as enhanced gut-tropism.

Importantly, our results confirmed the therapeutic potential of GSK-J4 as a promising treatment for inflammatory bowel diseases. In this regard, a recent study has shown that oral administration of GSK-J4 dampened gut inflammation in a mouse model of inflammatory colitis induced by DSS^5^. Mechanistic analyses in that study provided evidence of the GSK-J4 action on macrophages. The authors showed that JMJD3 inhibition exerted by the drug resulted in a reduced extent of the repressive mark H3K27 methylation in the promoter of the nuclear factor-erythroid 2-related factor 2 (Nrf2) gene, thus down-regulating its expression in macrophages. Of note, Nrf2 expression was required for the activation of the NLRP3 inflammasome, as the knockdown of Nrf2 dampened NLRP3 activation in macrophages stimulated by LPS or nigericin^5^. Despite that study provided evidence of a relevant role of GSK-J4 on macrophages during DSS-induced inflammatory colitis, our findings here revealed another relevant mechanism in the therapeutic activity of GSK-J4, which was not previously appreciated. In this regard, our findings show an important effect of GSK-J4 on regulating *raldh* transcription, yielding a significant increase in the RA synthesis by DCs, which consequently enhances their tolerogenic activity.

Emerging evidence has shown the involvement of JMJD3 and UTX demethylases not only in gut inflammation, but also in other inflammatory disorders such as rheumatoid arthritis, multiple sclerosis, sepsis, allergic asthma and diabetes. Accordingly, GSK-J4 exerts anti-inflammatory effects in different pathological conditions. For instance, this drug attenuates airways inflammation induced by house dust mite^25^ or by respiratory syncytial virus infection^26^. By targeting innate immune cells, GSK-J4 reduces the inflammatory cytokine storm in sepsis^27,28^ and ameliorates the development of central nervous system autoimmunity in a mouse model of multiple sclerosis^6^. Moreover, acting directly on target tissues involved in inflammatory disorders, such as joints in arthritis^29^ or pancreatic β-cells in diabetes^30^, GSK-J4 significantly reduces inflammation and disease manifestation in animal models of these disorders. Similarly, the inhibition of UTX activity in renal cells by GSK-J4 treatment ameliorated renal damage associated to diabetic kidney disease in a mouse model of type 2 diabetes^31^. Together these studies suggest that JMJD3 and UTX demethylases are master regulators of inflammation. Thereby, GSK-J4 seems to be a promising therapeutic drug with beneficial effects in a wide range of inflammatory disorders.

According to the broad spectrum of anti-inflammatory effects described for GSK-J4, the beneficial effects might be exerted by the action of the drug on different populations of inflammatory leukocytes. In this regard, the anti-inflammatory effect of GSK-J4 in macrophages not only occurs by the inhibition of Nfr2 gene expression and the consequent activation of the NLRP3 inflammasome^5^, but also by inducing the expression of the anti-inflammatory micro-RNA miR-146a^27^. On the other hand, the inhibition of the JMJD3 demethylase in neutrophils reduces the expression of the membrane proteinase 3, which is critical for IL-1β production, thus attenuating their inflammatory activity^28^. Moreover, GSK-J4 treatment strongly reduces the production of pro-inflammatory cytokines and decreases the expression of the machinery required for cytotoxic killing activity in human primary NK cells stimulated with IL-15^32^. Interestingly, the genetic *jmjd3* deficiency or the GSK-4-mediated pharmacologic inhibition of JMJD3 activity have a direct impact on CD4^+^ T-cells differentiation attenuating the acquisition of the Th17 phenotype^33^. Furthermore, by acting on DCs, the treatment with GSK-J4 attenuates the production of pro-inflammatory cytokines and increases the secretion of anti-inflammatory mediators, thus reducing the participation of the inflammatory lymphocytes Th1 and Th17 and promoting the generation of Treg cells in experimental autoimmune encephalomyelitis^6^. In addition to this latter GSK-J4 effect, in the present study we provided a deeper mechanistic insight showing that the drug exerts a strong increase in the RA production by DCs, inducing a shift from an immunogenic to a tolerogenic behaviour in these cells.

Importantly, Mucida and colleagues previously demonstrated that RA exerts an effect on naïve CD4^+^ T-cells differentiation, potentiating the generation of Treg induced by TGF-β and limiting the conversion into Th17 induced by TGF-β and IL-6^34^. The same authors demonstrated later that RA favoured the TGF-β-mediated Treg differentiation by directly affecting Treg, even in the absence of DCs^22^. Also, Hill and colleagues demonstrated that RA might contribute to Treg differentiation in vivo by dampening the inhibitory effect exerted by memory/effector CD4^+^ T-cells on the generation of Treg^35^. Moreover, we have observed here that RA acting on DCs might increase their ability to induce the conversion of naïve CD4^+^ T-cells into Treg in a RAR-dependent manner (Fig. S8). Probably all these RA-mediated mechanisms act together in vivo promoting the generation of peripheral Treg. Of note, we demonstrated here that the selective inhibition of the histone demethylase JMJD3/UTX in DCs induces upregulation of RALDH activity, increasing the RA production by these cells, which subsequently would impact in all three mechanisms described for RA-mediated Treg conversion.

## Materials and methods

### Animals

Six- to twelve-week-old mice of the C57BL/6 background were used for all experiments. Wild-type (WT; *Cd45.1*^−/−^ *Cd45.2*^+/+^) and congenic *Cd45.1*^+/+^ *Cd45.2*^−/−^ mice as well as *Foxp3*^*GFP*^ reporter mice were purchased from The Jackson Laboratory (Bar Harbor, ME). All mice were maintained and manipulated according to institutional guidelines at the pathogen-free facility after approval by the Ethical Review Committee of the Fundación Ciencia & Vida.

### Antibodies

Anti-CD3, anti-CD28, anti-CD16/32, anti-CD4-APCH7, anti-CD25-APC, PE anti-IL-17A, PECy7 anti-IFNγ, PeCy7 anti-CD45.1 were purchased from eBioscience (CA, USA). Anti-IL-4, anti-IFNγ, PE anti-α4β7, APC anti-CCR9, UV421 anti-IL-10 and APC or PE anti-CD11c were purchased from BioLegend (CA, USA). Anti-H3K4me3 and anti-H3 Abs were purchased from Abcam (Cambridge, UK). Anti-H3K27me3 and normal rabbit IgG Abs were purchased from Millipore (MA, USA)^6^.

### Flow cytometry

The expression of cell surface and intracellular markers on T cells and DCs were determined by FACS analysis after surface staining or intracellular staining with specific anti-mouse Abs (see the section “Antibodies”) as described before^36^. Briefly, for cytokine analysis on T cells, cells were stimulated for 4 h at 37 °C with 50 ng/mL PMA and 1 µg/mL ionomycin in the presence of 0.01 mg/mL BFA (Sigma-Aldrich). For intracellular staining, cells were first stained with Zombie Aqua (ZAq) Fixable Viability kit (Biolegend), followed by staining for cell-surface markers and then resuspended in fixation/permeabilization solution. After staining for surface markers, cells were fixed with 1% formaldehyde and permeabilized with 3% BSA and 0.5% saponin and further stained for intracellular proteins. All data were collected on a FACSCantoII (BD Biosciences) and analyzed with FACS Diva software (BD, New Jersey) or FlowJo software (TreeStar). ZAq^+^ cells were excluded of the analyses.

### Obtaining cells from spleen, MLN and colonic lamina propria

Spleen and MLN cells suspension were made by cutting the spleen or MLN with scissors, followed by digestion using 1 mg/mL Collagenase D (Roche) and 50 µg/mL DNase I (Roche) at 37 °C for 45 min. The cell suspensions were filtered through a 70-μm cell strainer (BD Biosciences), red blood cells lysed by the ammonium-chloride-potassium (ACK) buffer (Thermo Fisher) at RT for 5 min and the cells were resuspended in PBS + 10% FBS for further analysis. Colons were dissected, and the stool removed by washing two times with HBSS 1X without Ca^2+^ and Mg^2+^ and finally opened longitudinally. After that, the colon was cut into 0.5 cm pieces, washed with HBSS 1X without Ca^2+^ and Mg^2+^ and incubated in IEL medium (IMDM containing 2% FBS and 1 M HEPES) at 37 °C for 30 min, while constantly stirring. Colon pieces were washed using a 70-μm cell strainer, followed digestion using 1 mg/mL Collagenase D (Roche) and 0.25 mg/mL DNase I (Roche) in IEL medium at 37 °C for 45 min while constantly stirring. Digested tissue was passed through a 70-μm cell strainer obtaining a single cell suspension that was subjected to centrifugation in a Percoll gradient (67%/44%). Mononuclear cells were removed from the interphase and resuspended in culture medium for further analysis.

### Cell isolation and in vitro T-cell differentiation

Dendritic cells from spleens of WT (C57BL/6) mice were enriched by positive selection using the MACS purification kit based in microbeads coupled to anti-CD11c Ab (Miltenyi Biotec). To avoid initial contamination with Foxp3^+^ CD4^+^ T cells, we isolated naïve CD4^+^ T cells from the spleens of *Foxp3*^*GFP*^ reporter mice^37^. Splenic CD4^+^ T cells from *Foxp3*^*GFP*^ mice were enriched by negative selection using the CD4 isolation kit II (Miltenyi Biotec) following the manufacturer’s instructions and further purified by sorting for CD4^+^ CD25^−^ Foxp3^−^ cells using a FACS ARIA II cell sorter (Becton Dickinson, NJ, USA). For polarizing experiments, naïve T cells were stimulated with DCs or with plate-bound anti-CD3 and soluble anti-CD28 in the presence or absence of 25 nM GSK-J4 (Tocris Bioscience). For DCs-free stimulation, U-bottom plates were coated with 10 μg/mL of anti-CD3 diluted in PBS, and 1 µg/ml soluble anti-CD28 was added to the cultures as described before^38^. For DCs stimulation, DCs were first incubated with GSK-J4 25 nM or vehicle (0.1% ethanol) for 18 h, then washed and cultured for 5 days with naïve T cells at a 1:5 ratio in the presence of 1 μg/mL anti-CD3. Cultures under iTreg standard polarizing conditions contained 5 ng/mL TGF-β, 100 U/mL IL-2, 10 μg/mL anti-IL-4 and 10 μg/mL anti-IFN-γ. When indicated, in addition to the standard polarizing conditions, 10 nM RA, 25 nM GSK-J4 or both were added to the culture medium.

### Treg-Th17 transdifferentiation assay

Naïve CD4^+^ CD25^−^ GFP^−^ T cells were isolated from *Foxp3*^*GFP*^ mice by cell-sorting and iTreg were generated as described above. CD4^+^ GFP^+^ iTreg were subsequently purified by cell-sorting and cultured (10^5^ cells/well) with DCs (2 × 10^4^ cells/well) in the presence of anti-CD3 Ab (1 μg/mL) in U-bottom 96-well plates in IMDM medium containing 2% FBS and 1 M HEPES at 37 °C and 5% CO_2_. Subsequently, cells were cultured in the presence of Th17 biased conditions: TGF-β1 (5 ng/mL), IL-6 (20 ng/mL), IL-1β (10 ng/ml), anti-IL-4 (5 μg/mL) and anti-IFNγ (5 μg/mL) for 5 days. Treg stability and transdifferentiation towards Th17 lineage were assessed by the quantification of Foxp3 and IL-17A expression by flow cytometry.

### In vitro suppression assays

Naïve CD4^+^ T-cells isolated from *Foxp3*^*gfp*^ mice were differentiated to iTreg in the presence of DCs (treated or not with GSK-J4) as indicated above. After 4 days of incubation, iTreg (GFP^+^ CD4^+^) were isolated by cell sorting. CD4^+^ CD25^−^ naïve T-cells were loaded with 5 μM CTV and then cultured (5 × 10^4^ cells/well) with DCs (10^4^ cells/well) in the presence of anti-CD3 Ab (1 μg/mL) and increasing amounts of iTreg (Teff:Treg ratios 2:1, 1:1 and 1:2) in U-bottom 96-well plates. Three days later, the extent of Teff proliferation was assessed in the CD4^+^ CTV^+^ population by flow cytometry.

### Aldefluor assay

RALDH activity in DCs was measured using ALDEFLUOR staining kits (StemCell Technologies), according to the manufacturer’s protocol. Briefly, 10^6^ DCs/mL were resuspended in ALDEFLUOR assay buffer containing Aldefluor reagent (1.5 µM final concentration) in the presence or absence of DEAB (diethylaminobenzaldehyde, 15 µM) and then DCs were incubated 45 min at 37 °C. After Aldefluor staining, the cells were incubated for 30 min at 4 °C with APC-conjugated anti-CD11c. ALDEFLUOR-reacted cells were analysed by flow cytometry using a FACS Canto II cytometer (BD), and data were analysed using FACS Diva software (BD).

### Analysis of RALDH isoenzymes mRNA production by real-time RT-PCR

CD11c^+^ splenic DCs from C57BL/6 mice were either left unstimulated or stimulated with 100 ng/ml LPS for 16 h in the presence or absence of 25 nM GSK-J4. Cells were lysed, the RNA was extracted and cDNA was generated as described before^39^. A 10-µl real-time PCR reaction included 2.5 µl cDNA, 5 µl KAPA SYBR FAST (KAPA Biosystems), and 500 nM primers and water as indicated by the manufacturer’s instructions. PCR was carried out for 40 cycles with 95 °C melting (30 s), 61 °C (18S RNA) or 63 °C (*raldh1, raldh2, and raldh3*) annealing (45 s), and 72 °C extension (40 s). All reactions were performed on a rotor Gene Q (Qiagen). Primer sequences were as follows: RALDH1 forward, 5′-GAC AGG CTT TCC AGA TTG GCT-3′; RALDH1 reverse, 5′-AAG ACT TTC CCA CCA TTG AGT G-3′; RALDH2 forward, 5′-CAG AGA GTG GGA GAG TGT TCC-3′; RALDH2 reverse, 5′-CAC ACA GAA CCA AGA GAG AAG G-3′; RALDH3 forward, 5′-CAC AGG CTC CAT TTG GTG G-3′; RALDH3 reverse, 5′-TGT CCA GCT TTT GAG GAA GAA G-3′; 18S RNA forward, 5′-GAG GGA GCC TGA GAA ACG GC-3′; 18S RNA reverse, 5′-CGG GTC GGG AGT GGG TAA TTT-3′. For relative quantification, mRNA expression in each sample was normalized by comparison with the 18S RNA mRNA expression using the − ΔΔCt method as previously described^40^.

### Chromatin immunoprecipitation and real time PCR

After cell sorting, CD11c^+^ DCs were fixed with 1% formaldehyde for 10 min and ChIP followed by real time PCR were performed as described before^6^. Primers for the RALDH1, RALDH2, and RALDH3 promoters were as follows: RALDH1P forward, 5′-TCC TTC AAG GTC TGT GAC CAA AGC-3′; RALDH1P reverse, 5′-AAC AGG GAC CTG AGG AGT GTG TTT-3′; RALDH2P forward, 5′-GGT GTG GAT GGG AAG AGG AAA GGA AA-3′; RALDH2P reverse, 5′-CAT CTG CCT TGG GTT GCC TGG ATA TT-3′; RALDH3P forward, 5′-GCA GGA AAC TTC CGT CAC AC-3′ and RALDH3P reverse 5′-AGG AAA CTT CCG TCA CAC CC-3′. PCR was carried out for 40 cycles with 95 °C for 10 s (melting), 59 °C for 5 s (annealing) and 72 °C for 5 s (extension).

### Colitis induced by dextran sulfate sodium (DSS) and evaluation

Acute DSS colitis was induced in C57BL/6 mice according to the previously published method with minor modification^41^. Mice composing the DSS or the DSS + GSK-J4 groups were fed 1.5% (w/v) DSS (MW: 36–50 kDa, MP Biomedicals) dissolved in the drinking water on day one. Fresh DSS solution was provided every second day. In addition, mice received i.p. injections of vehicle or 0.5 mg/kg GSK-J4^6^ at different times as indicated below. Disease symptoms of colitis were assessed daily by measurement of bodyweight, evaluation of stool consistency and detection of bloody stools. Clinical score was scored using the following parameters: 0, normal faeces and no blood; 1, soft faeces and no blood; 2, very soft faeces and blood traces; 3, colitis and blood. In other acute colitis experiments, 10^6^ bone marrow-derived vehicle-treated or GSK-J4-treated DCs from WT mice were transferred i.v. into WT C57BL/6 recipient the same day that begins the acute colitis experiment.

### Ex vivo colon slices culture and Cytometric bead array

One cm^2^ colon tissue were cultured in 1 mL IMDM medium during 24 h at 37 °C and 5% CO_2_. Supernatants were collected from ex vivo colon slices culture and store at − 80 °C until further analysis by CBA. Cytokine production was analyzed using the Mouse Th1/Th2/Th17 Cytokine Kit following the manufacturer’s instructions (BD Biosciences).

### Generation of DCs

Bone marrow-derived DCs precursors from WT mice were prepared as previously described^42^. Briefly, DCs precursors were grown in RPMI 1640 medium (Hyclone, Logan, UT) supplemented with 5% heat-inactivated FBS (Biological Industries, Beit Haemek, Israel) and 10 ng/ml recombinant mouse GM-CSF (PeproTech, Rocky Hill, NJ). Differentiation of DCs was routinely assessed obtaining ≥ 90% CD11c^+^ cells. In some experiments, day 5 DCs were pulsed either with DMSO (vehicle-treated DCs) or with 10 nM GSK-J4 (GSK-J4-treated DCs) for 18 h, washed and used for further experiments.

### Statistical analysis

Data are presented as mean ± SEM and *P* values were analyzed using the Student’s t-test or one-way ANOVA followed by Dunnett’s test using Prism (GraphPad software) as described before^6^. In some cases, *P* values were analyzed from Mann–Whitney rank sums two-tailed *U*-test by using GraphPad Prism software. ns, no significant. Significant values were expressed as *p < 0.05; **p < 0.01; ***p < 0.001.

## Supplementary Information

Supplementary Figures.

## Data Availability

The datasets generated during and/or analysed during the current study are available from the corresponding author on reasonable request.

## Abbreviations

Ab: Antibody
BFA: Brefeldin A
BSA: Bovine serum albumin
CCR9: C–C chemokine receptor 9
cLP: Colonic lamina propria
DC: Dendritic cell
DEAB: Diethylaminobenzaldehyde
DSS: Dextran sodium sulphate
FACS: Fluorescence-activated cell sorting
Foxp3: Forkhead box P3
GALT: Gut-associated lymphoid tissues
GFP: Green fluorescent protein
GM-CSF: Granulocyte-monocyte colony stimulating factor
GSK-J4: GlaxoSmithKline J4
H3K4me3: Histone H3 trimethylated at the lysine 4
H3K27me3: Histone H3 trimethylated at the lysine 27
IBD: Inflammatory bowel diseases
IFN-γ: Interferon gamma
IL-*n*: Interleukine *n*
JMJD3: Jumonji demethylase 3
LPS: Lipopolysaccharide
MLN: Mesenteric lymph nodes
PMA: Phorbol 12-myristate 13-acetate
RA: Retinoic acid
RALDH: Retinaldehyde dehydrogenase
TGF-β: Transforming growth factor beta
Th*n*: T helper *n*
Treg: Regulatory T cell
UTX: Ubiquitously transcribed tetratricopeptide repeat, X chromosome
WT: Wild-type
