# Supplementary material for: The demethylase inhibitor GSK-J4 limits inflammatory colitis by promoting de novo synthesis of retinoic acid in dendritic cells
Source: Sci Rep. 2021 Jan 14;11:1342. doi: 10.1038/s41598-020-79122-3 (PMC7809056; doi:10.1038/s41598-020-79122-3)
Supplement: Supplementary file 1 — Supplementary Figures. [file 41598_2020_79122_MOESM1_ESM.pdf]

SUPPLEMENTARY MATERIAL

The demethylase inhibitor GSK-J4 limits inflammatory colitis by promoting *de novo* synthesis of retinoic acid in dendritic cells

Cristian Doñas, Jocelyn Neira, Francisco Osorio-Barrios, Macarena Carrasco, Dominique Fernández, Carolina Prado, Alejandra Loyola, Rodrigo Pacheco and Mario Rosemblatt

Figure S1

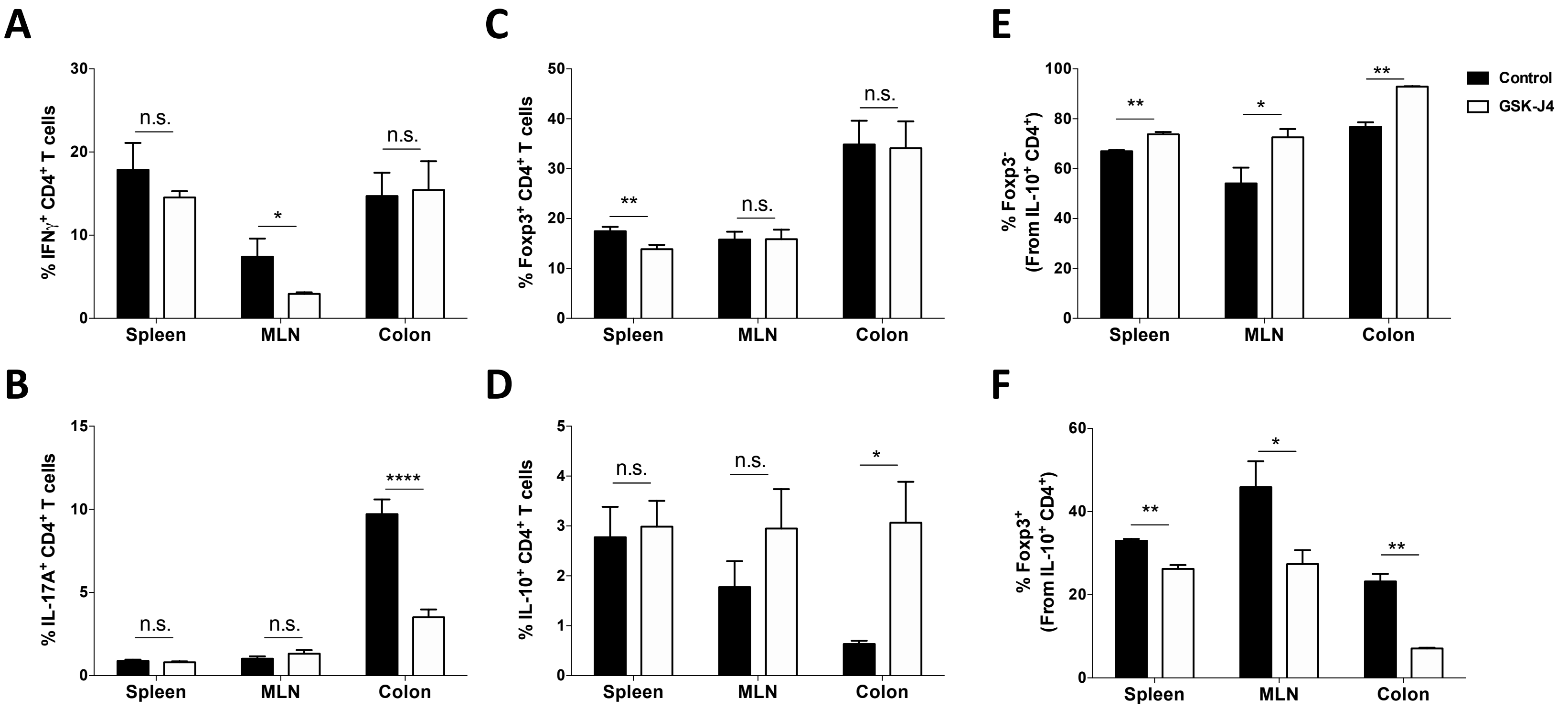

Figure S1. Systemic GSK-J4 treatment decreases IL17A$^+$ CD4$^+$ T-cells and increases IL-10$^+$ CD4$^+$ T-cells in the colon. Mice were treated as described in figure 1A and, at the peak of colitis severity (day 12), mice were sacrificed and mononuclear cells were isolated from spleen, MLN and colon followed by *ex vivo* stimulation with PMA/ionomycin in the presence of brefeldin A. Intracellular cytokine staining analysis were carried out by flow cytometry. Frequency of (**A**) IFNγ$^+$, (**B**) IL-17A$^+$, (**C**) Foxp3$^+$, and (**D**) IL-10$^+$ cells from CD4$^+$ T-cells. The percentage of Foxp3$^-$ (**E**) and Foxp3$^+$ (**F**) cells from the IL-10$^+$ CD4$^+$ gate were also evaluated. Values represent the average percentage ± SEM from at least seven mice per group. *, $p<0.05$; **, $p<0.01$; ****, $p<0.0001$; as determined by Student's t-test. n.s., no significant.

Figure S2

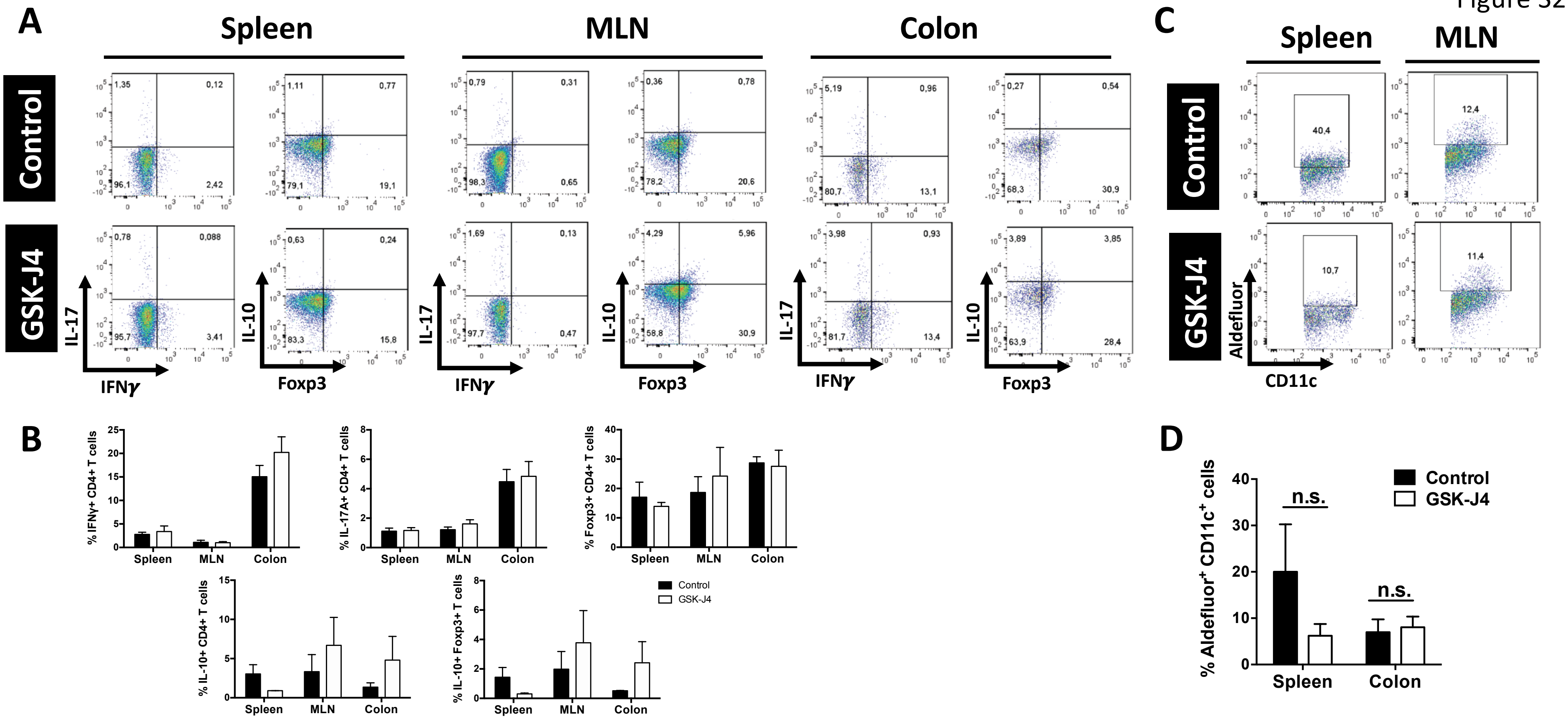

Figure S2. Analysis of T cell phenotype at day 8 upon GSK-J4 treatment in mice exposed to DSS. Mice were treated with DSS in the presence of GSK-J4 or vehicle as indicated in the figure 1A. Mice were sacrificed at day 8 and mononuclear cells were isolated from spleen, MLN and colon followed by *ex vivo* stimulation with PMA/ionomycin in the presence of brefeldin A. Intracellular cytokine staining analysis were carried out in the $CD4^{+}$ ZAq^{-} gate by flow cytometry. (**A**) Representative dot plots are shown. The percentage of cells in each quadrant is indicated. (**B**) Quantification of the percentage of Th1 ($IFN\gamma^{+}$), Th17 ($IL\text{-}17A^{+}$), Treg ($Foxp3^{+}$), $IL\text{-}10^{+}$ T $CD4^{+}$ cells and $IL\text{-}10^{+}$ Treg ($IL\text{-}10^{+}$ $Foxp3^{+}$) cells. (C-D) RALDH activity was analysed by Aldefluor assay in $CD11c^{+}$ cells isolated from the spleen or MLN of mice treated with GSK-J4 or vehicle (control) that received DSS for 8 days. (**C**) representative dot-plots. (**D**) quantification of the frequency of $Aldefluor^{+}$ cells in the $CD11c^{+}$ ZAq^{-} gate. Values represent mean ± SEM from three mice per group. No significant differences were found by Student's t-test.

Figure S3

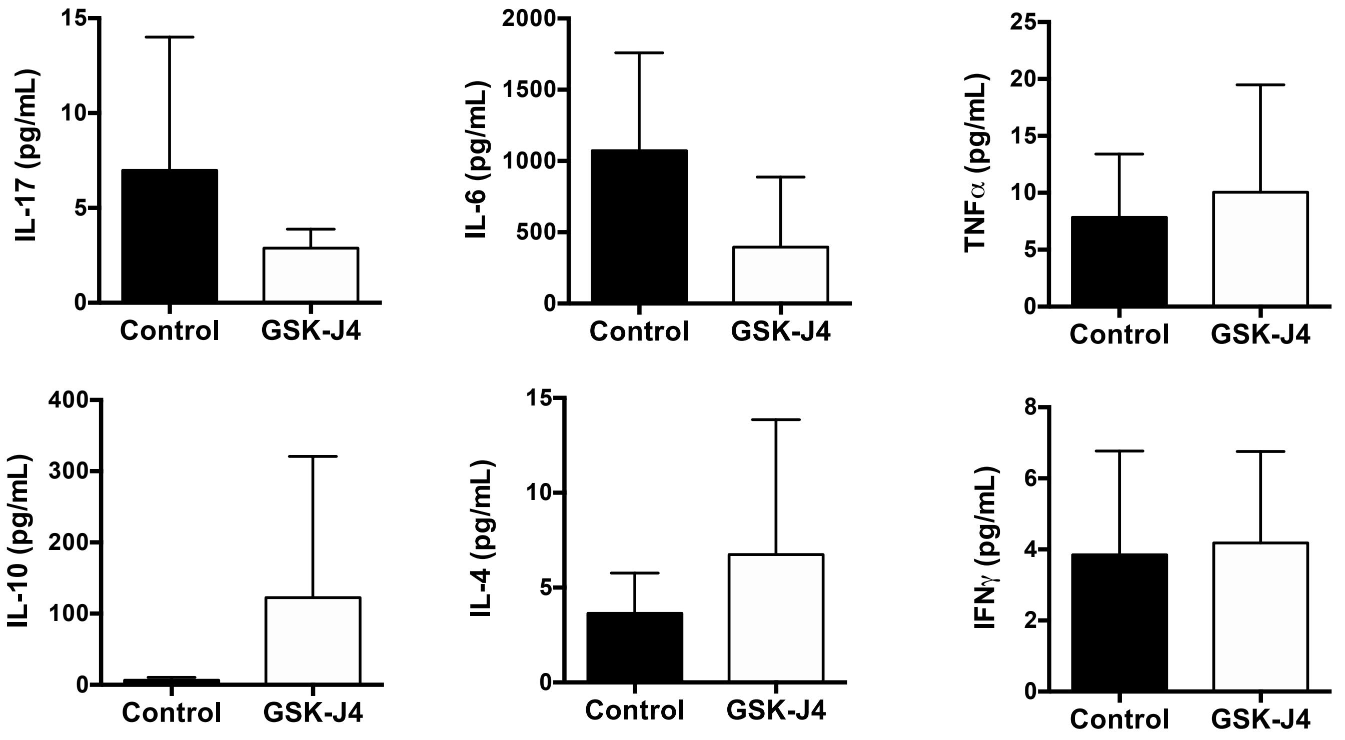

Figure S3. Analysis of colonic cytokines at day 8 upon GSK-J4 treatment in mice exposed to DSS. Mice were treated with DSS in the presence of GSK-J4 or vehicle as indicated in the figure 1A. Mice were sacrificed at day 8 and colon slices were cultured in fresh medium for 24 h and the supernatant was evaluated for the secretion of IL-17, IL-6, TNFα, IL-10, IL-4 and IFNγ by CBA. Values represent mean ± SEM from 7 (IL-17, IL-6, TNFα), 3-5 (IL-10, IFNγ) or 6 (IL-4) mice per group. No significant differences were found by Student's t-test.

Figure S4

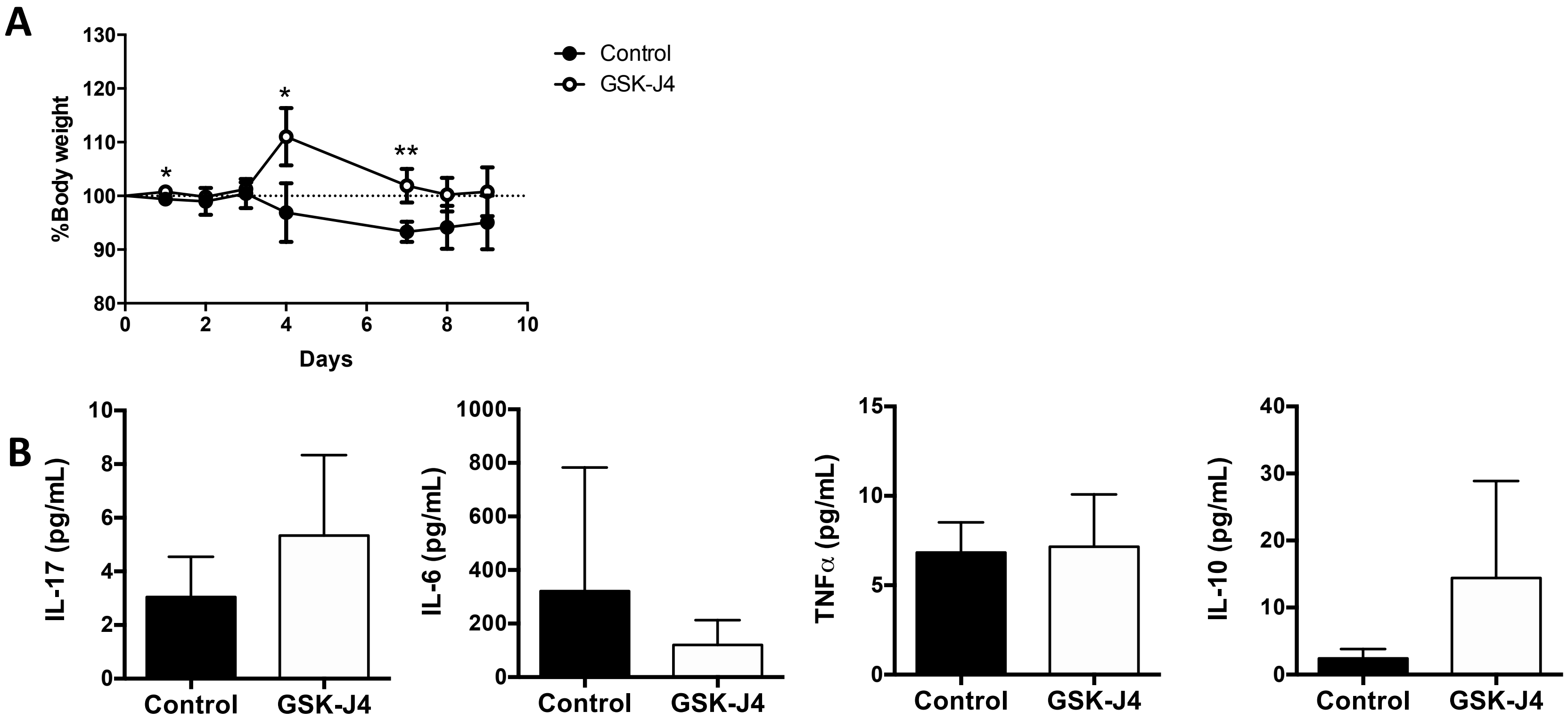

Figure S4. GSK-J4 treatment attenuates the inflammation in mice deficient on adaptive immune system upon DSS-induced colitis. Rag1$^{-/-}$ mice were treated with DSS in the presence of GSK-J4 or vehicle as indicated in the figure 1A. (**A**) The percentage of body weight change was determined along the time-course of disease development. (**B**) Mice were sacrificed at day 9 and colon slices were cultured in fresh medium for 24 h and the supernatant was evaluated for the secretion of IL-17, IL-6, TNFα and IL-10 by CBA. (A-B) Values represent mean ± SEM from 4 mice per group. *, $p<0.05$; **, $p<0.01$ by Student's t-test.

Figure S5

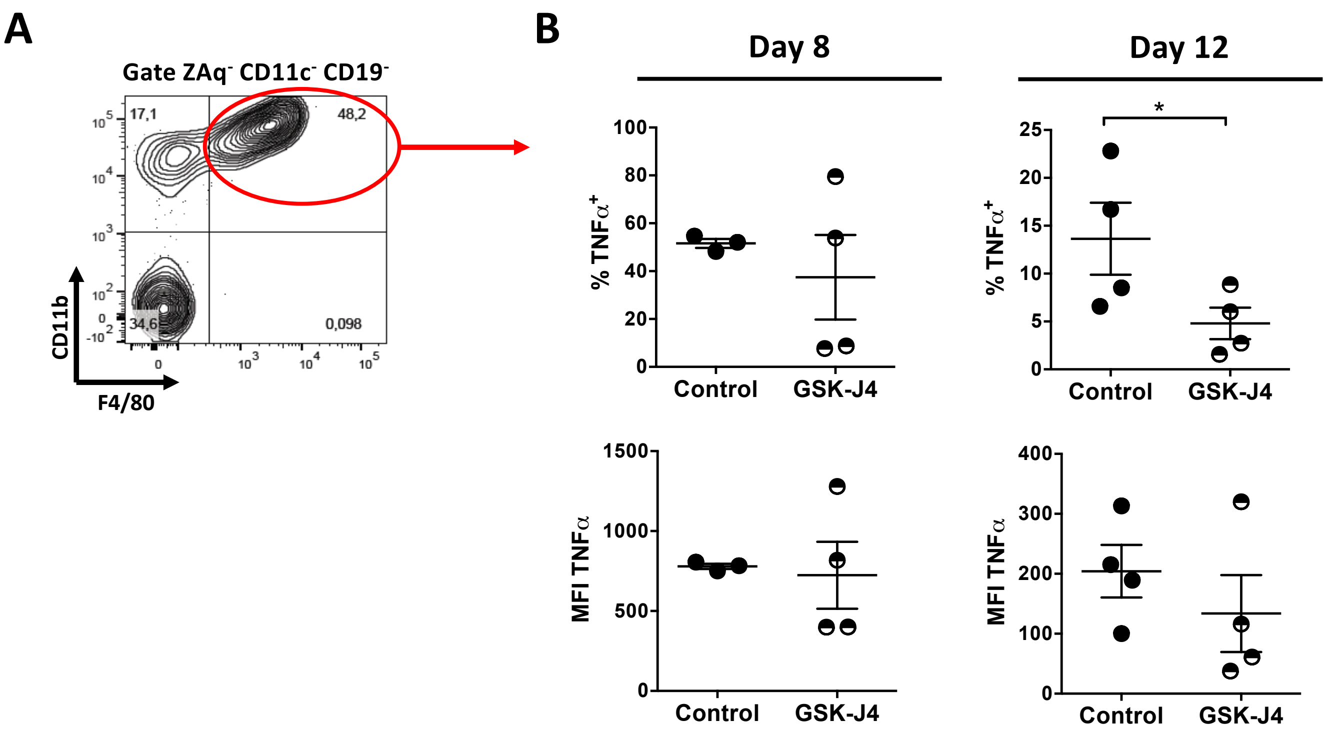

Figure S5. GSK-J4 treatment reduces TNFα production by macrophages upon DSS-induced colitis. Wild-type mice were treated with DSS in the presence of GSK-J4 or vehicle as indicated in the figure 1A. Mice were sacrificed at day 8 or 12 and peritoneal macrophages were isolated followed by *ex vivo* stimulation with PMA/ionomycin in the presence of brefeldin A. Intracellular TNFα staining analysis was carried out in the $CD11c^-$ $CD19^-$ $CD11b^+$ $F4/80^+$ ZAq^- gate by flow cytometry. (**A**) Representative dot-plot showing CD11b versus F4/80 immunostaining in the $CD11c^-$ $CD19^-$ ZAq^- gate. TNFα analysis was performed in the population indicated inside the red ellipse. (**B**) Quantification of TNFα production as the frequency of TNFα^+ cells (top panels) or the MFI associated to TNFα immunostaining (bottom panels) at day 8 (left panels) or day 12 (right panels) after colitis induction. Each symbol represents data obtained from a single mouse (n = 4 per group). Mean ± SEM are shown. *, $p<0.05$ by Student's t-test.

Figure S6

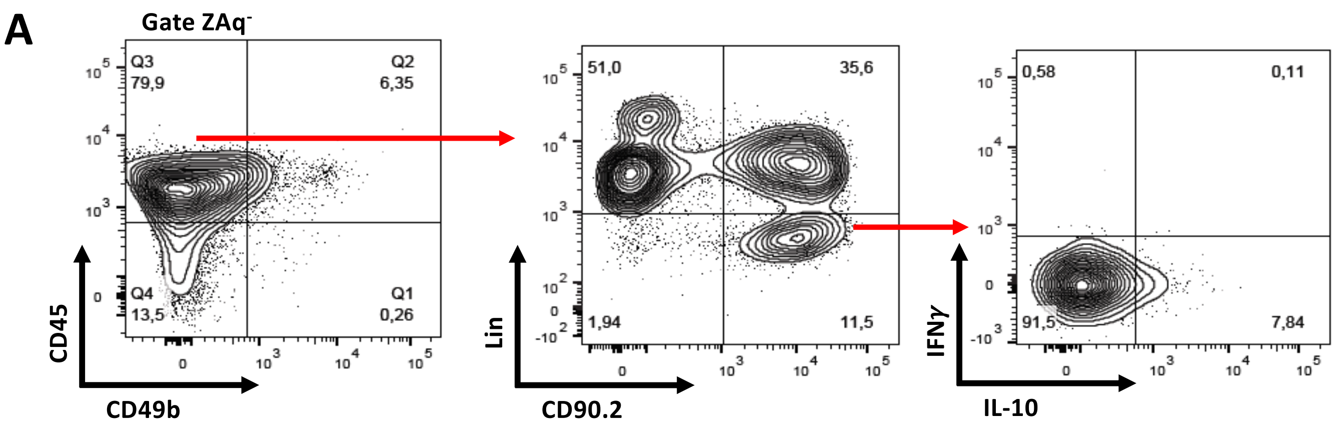

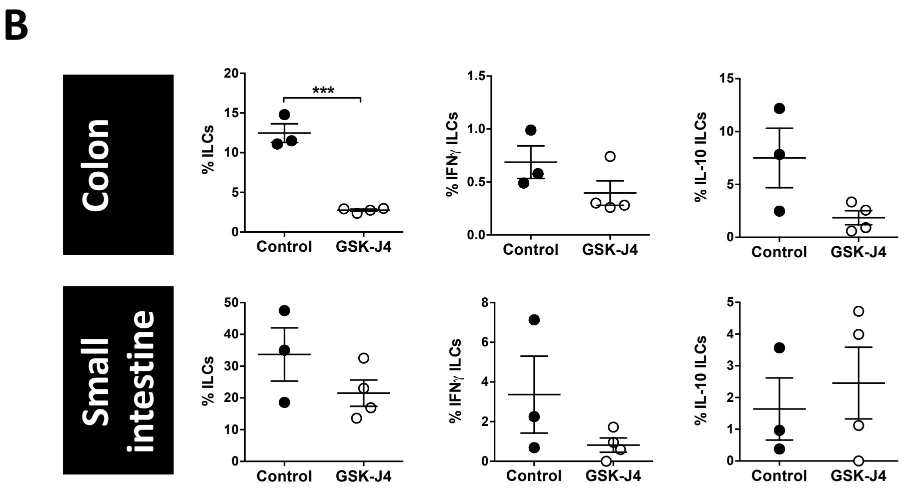

Figure S6. GSK-J4 treatment reduces the frequency of colonic ILCs upon DSS-induced colitis. Wild-type mice were treated with DSS in the presence of GSK-J4 or vehicle as indicated in the figure 1A. Mice were sacrificed at day 12 and mononuclear cells were isolated from colonic lamina propria or from small intestine lamina propria followed by *ex vivo* stimulation with PMA/ionomycin in the presence of brefeldin A. Intracellular cytokine staining analysis were carried out in the Lineage$^-$ CD90.2$^+$ CD49b$^-$ CD45$^+$ ZAq$^-$ gate by flow cytometry. (**A**) Representative dot-plot showing the gating strategy. (**B**) Quantification of the frequency of total ILCs (left panels) and the percentage of ILCs producing IFN***γ*** (middle panels) and IL-10 (right panels) in the colon (top panels) or small intestine (bottom panels). Each symbol represents data obtained from a single mouse (n = 3-4 per group). Mean ± SEM are shown. *, p<0.05 by Student's t-test.

Figure S7

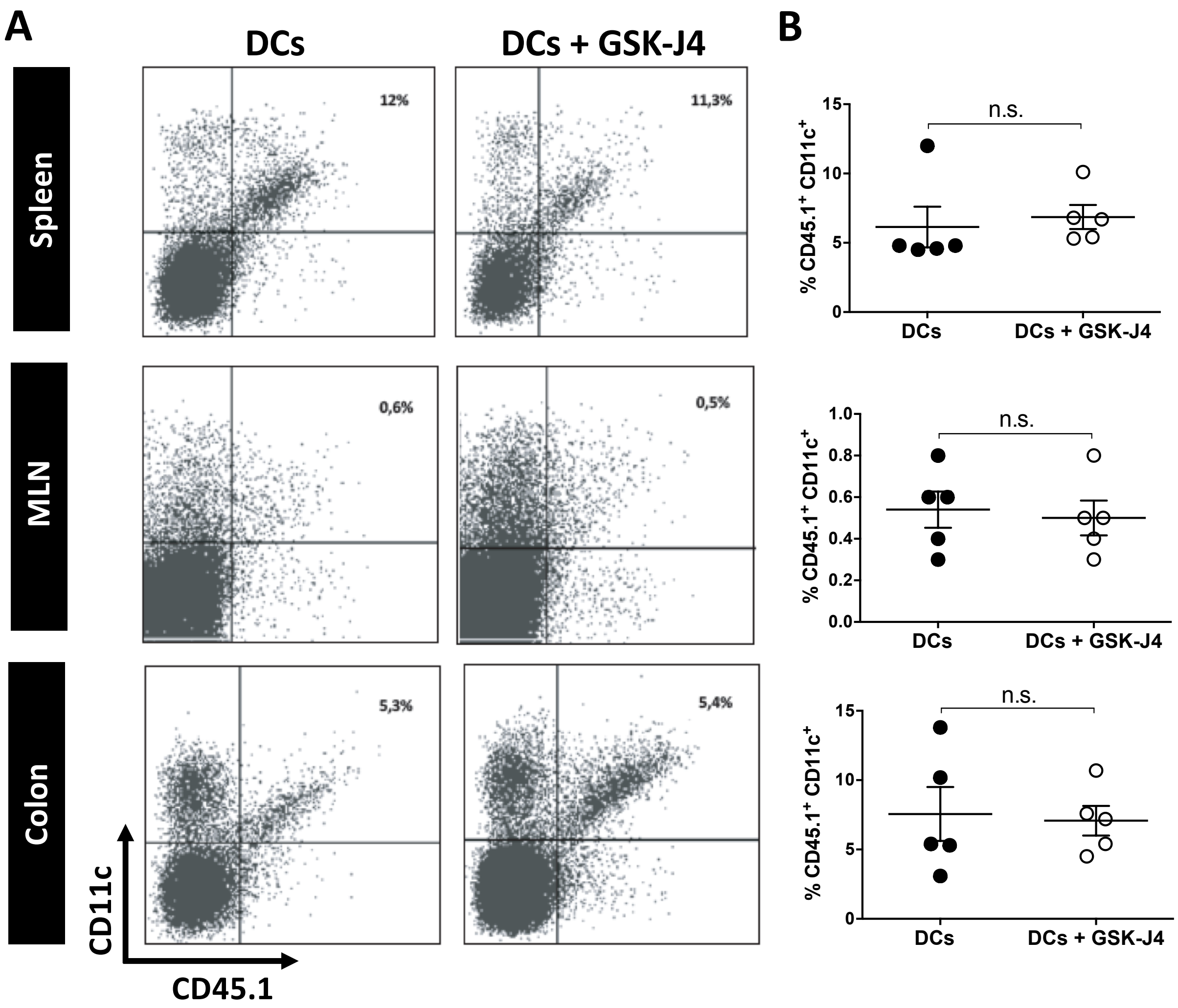

Figure S7. Ex vivo GSK-J4 treated DCs does not have a preferential intestine migration pattern. Bone marrow-derived DCs obtained from *Cd45.1*$^{+/+}$ *Cd45.2*$^{-/-}$ mice were treated with vehicle or with 25 nM GSK-J4 and then 3x10^{6} DCs/mice were i.v. transferred into *Cd45.1*$^{-/-}$ *Cd45.2*$^{+/+}$ wild-type recipient mice on the same day that DSS treatment began. DSS administration was maintained for 6 days and mice were sacrificed at day 9 as indicated in figure 2A. (**A**) Representative dot-plots of CD11c^{+} CD45.1^{+} DCs in spleen, MLN and colonic lamina propria (Colon). Numbers represent the frequency of cells in the corresponding quadrant. (**B**) Frequency of CD11c^{+} CD45.1^{+} cells in the spleen, MLN, and cLP from mice treated with DCs/vehicle and DCs/GSK-J4. Each symbol represents data obtained from a single mouse (n = 5 per group). Mean ± SEM are shown. Differences were evaluated by Student's t-test. n.s., no significant results were found.

Figure S8

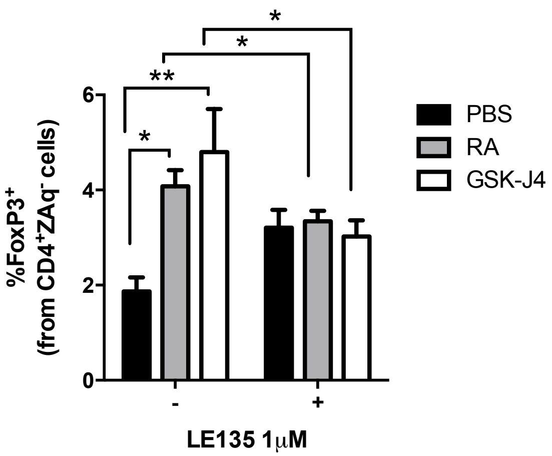

Figure S8. Treg differentiation induced by GSK-J4 is dependent on the RAR stimulation on DCs. BMDCs were treated with PBS, RA (10 nM) or GSK-J4 (25 nM) in the presence or in the absence of the RAR antagonist LE135 (1μM), then washed and co-cultured with naive $CD4^+$ T-cells in the presence of anti-CD3 Ab (1 μg/mL) for 5 days. The extent of Treg differentiation was evaluated as the percentage of $Foxp3^+$ cells in the $CD4^+$ ZAq^- gate. Values are mean with SEM from three independent determinations per group. *, $p<0.05$; **, $p<0.01$ by two-way ANOVA followed by Tukey's post-hoc multiple comparison test.

Figure S9

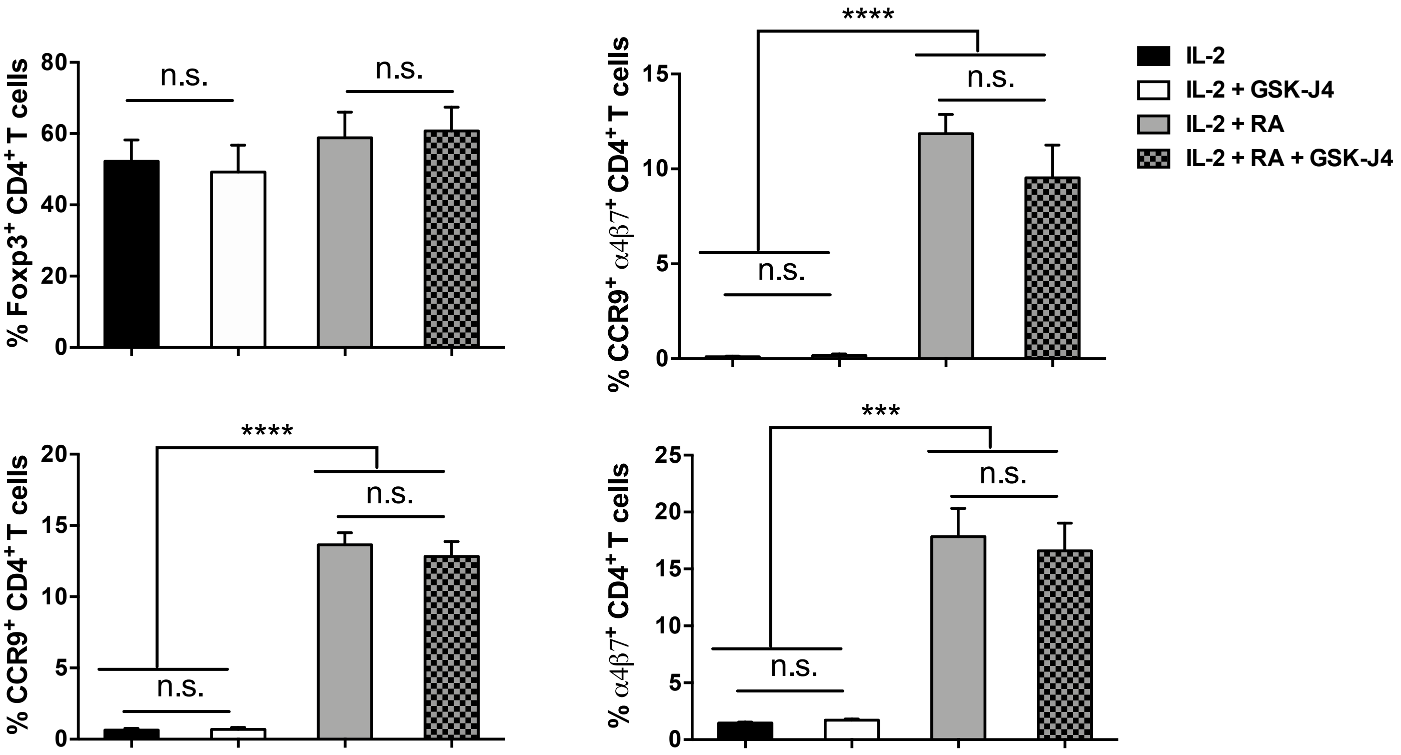

Figure S9. GSK-J4 does not directly affect Foxp3, CCR9 and α4β7 expression on CD4⁺ T cells. Naive $CD4^+$ $CD25^-$ $Foxp3^-$ T cells were isolated from *Foxp3*^{GFP} mice by cell-sorting and activated with anti-CD3 and anti-CD28 Abs under standard Treg polarising conditions (IL-2), or in the presence of GSK-J4 25 nM, RA 10 nM or both together for 4 days. The extent of iTreg generation was evaluated as the frequency of GFP^+ ($Foxp3^+$) cells in the $CD4^+$ population (top left panel). CCR9 and α4β7 expression were also evaluated. The quantification of the frequency of $CCR9^+$ $α4β7^+$ (top right panel), $CCR9^+$ (bottom left panel) and $α4β7^+$ (bottom right panel) $CD4^+$ T cells are shown. Data represent mean ± SEM from six independent experiments. ***; $p < 0.001$; ****, $p<0.0001$ as determined by one-way ANOVA followed by Tukey's post-hoc test. n.s., not significant differences were found.
